# Bayesian analyses of direct radiocarbon dates reveal geographic variations in the rate of rice farming dispersal in prehistoric Japan

**DOI:** 10.1126/sciadv.adc9171

**Published:** 2022-09-21

**Authors:** Enrico R. Crema, Chris J. Stevens, Shinya Shoda

**Affiliations:** ^1^ Department of Archaeology, University of Cambridge, Cambridge, UK.; ^2^ McDonald Institute for Archaeological Research, University of Cambridge, Cambridge, UK.; ^3^ Nara National Research Institute for Cultural Properties, Nara, Japan.; ^4^ BioArCh, University of York, York, UK.

## Abstract

The adoption of rice farming during the first millennium BC was a turning point in Japanese prehistory, defining the subsequent cultural, linguistic, and genetic variation in the archipelago. Here, we use a suite of novel Bayesian techniques to estimate the regional rates of dispersal and arrival time of rice farming using radiocarbon dates on charred rice remains. Our results indicate substantial variations in the rate of dispersal of rice within the Japanese islands, hinting at the presence of a mixture of demic and cultural diffusion, geographic variations in the suitability of its cultivation, and the possible role of existing social networks in facilitating or hindering the adoption of the new subsistence economy.

## Teaser

The adoption of rice farming in prehistoric Japan was characterized by regional episodes of slowdowns and accelerations.

## INTRODUCTION

The dispersal of agriculture, its timing, speed, and the mechanisms behind its spread have long been seen as one of the most important shifts that laid the genetic, linguistic, and cultural foundations for many regions of the world (*1*, *2*). Reconstructing details and variation in this process has been a key research agenda for nearly a half-century of archaeological, linguistic, and genetic research, with much emphasis dedicated to the mode (e.g., demic versus cultural diffusion) and tempo (i.e., estimates of arrival dates and the pace of the dispersal process) of this key process. The primary mode of diffusion has been inferred from genetic and archaeological evidence, with demic diffusion typically characterized by a geographic gradient in gene frequency surfaces along the main direction of dispersal, signaling the admixture of migrant farmers and local hunter-gatherers (*3*). While this relationship portrays a continental-scale process of agricultural diffusion common to many parts of the world, closer examination of the archaeological evidence has increasingly revealed that the tempo of this process can vary substantially across different areas, with substantial episodes of local slowdowns and accelerations (*4*–*6*). These variations in expansion rates have been explained by several hypotheses, ranging from the environmental suitability of specific farming practices to the possibility that different modes of diffusion were locally dominant (*4*, *6*–*13*). For example, on the basis of a reaction-diffusion model, Fort (*14*) argued that a mixed demic-cultural diffusion model is expected to be faster than a purely demic diffusion model, while a purely cultural diffusion model would lead to the slowest dispersal rates. He then identified putative regions of dominant modes in different parts of Europe using observed variation in the dispersal rate of farming.

While the extent to which the front speed of dispersal alone can reveal the dominant mode of agricultural diffusion remains an open question, it is undeniable that accurate and precise estimates of dispersal rates are fundamental steps for determining the underlying processes of agricultural diffusion. The increasing availability of large collections of radiocarbon dates provides robust empirical foundations to undertake this research agenda. However, most studies typically cover vast geographic scales, use mixed-quality samples (e.g., short-lived samples of carbonized seeds versus culture chronologies), and do not fully account for the different sources of uncertainties that characterize the archaeological record. There are some notable exceptions (*15*, *16*), but to our knowledge, a comprehensive study aiming to detect variations in the tempo of the dispersal processes within smaller geographical regions, exclusively using direct dates from seeds, and providing a more robust inferential framework to discern genuine signals from statistical artifacts does not exist.

Here, we contribute to this wider research agenda by analyzing regional variations in the dispersal of rice farming in Japan during the first millennium BC. The high intensity of archaeological excavations and comparatively large number of radiocarbon dates on charred remains, combined with the diverse ecological and environmental settings of the Japanese islands, make this region highly suited for investigating the drivers behind the uneven dispersal rates of agriculture.

**Fig. 1.**
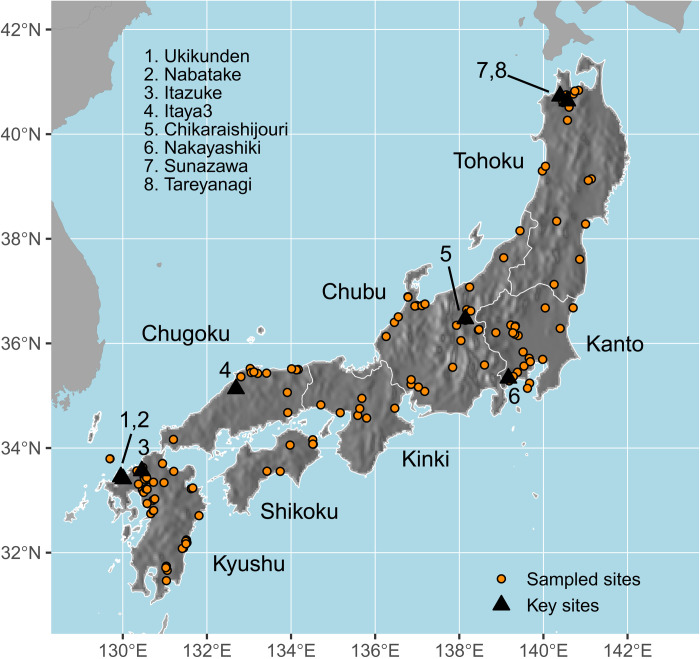
Maps of study area. Distribution map of archaeological sites with charred rice remains (*n* = 132) used for analyses and locations of key sites mentioned in the text.

The arrival of rice and millet (foxtail and broomcorn) agriculture in the Japanese archipelago is traditionally used as the defining feature that marks the end of the Jomon period and the beginning of the Yayoi period (*17*, *18*). The former period is associated with a subsistence economy largely based on hunting, fishing, and gathering, with a smaller contribution of small-scale plant husbandry (*19*, *20*), whereas the Yayoi period is associated with the introduction of a cultural package of continental origin that is archaeologically associated with paddy fields, farming tools, moated settlements, new types of pottery, dwellings, burials, and, at a later stage, metallurgy (*18*). This continental package was brought into the Japanese islands by migrant communities from the Korean peninsula (*21*, *22*) to the northern coastal area of the southwest island of Kyushu (Fig. 1) during the first millennium BC (*23*) and subsequently dispersed in the rest of the Japanese archipelago. This process took several centuries, effectively determining a different timing of the economic transition in different parts of the Japanese islands (*24*, *25*). While both millets and rice have continental origins, the extent to which their arrival and dispersal within the Japanese archipelago were synchronous is still an open question, limited by the comparatively smaller radiocarbon evidence for the former. Here, we concentrate specifically on the dispersal of rice agriculture.

Several authors have suggested that the pace of the diffusion of rice agriculture in Japan was geographically uneven, hinting at the possibility of different forms of interaction between migrant communities and incumbent hunter-gatherers. While details vary substantially (*15*, *24*, *26*–*29*), the general consensus sees the dispersal of rice farming originating from northern Kyushu (*28*), initially limited to three of the four main islands of the Japanese archipelago (Kyushu, Shikoku, and Honshu; Fig. 1) until later historical periods (*30*, *31*), and characterized by faster dispersal rates in the west and a significant slowdown in the east.

These general patterns broadly mirror different population densities of the incumbent population (*32*), regional social networks inferred from pottery styles (*33*), and latitudinal differences that might correlate to differences in the suitability of rice farming. However, a formal evaluation of these factors is currently hindered by limitations in the data and the methodologies used. First, the presence of a radiocarbon calibration plateau at around 2500 ^14^C BP yields calibrated dates spanning a substantial portion of the eighth to fifth century BC, effectively limiting accurate inferences about the timing of the dispersal within western Japan. Second, most studies have relied on a mixture of direct dates on carbonized seeds, dates contextually and stratigraphically associated with farming (e.g., samples recovered from paddy field layers), and ceramic typology. While this offers larger sample sizes and multiple proxies for the presence of farming at a given location, they are severely limited in their capacity to provide accurate chronological estimates that formally account for different sources of uncertainty. The most notable example of this problem is the use of pottery-based chronologies, characterized by major discrepancies between scholars with regard to the start and end date of key phases (*28*, *34*, *35*). Third, with one notable exception (*15*), claims on the arrival dates in different regions have been based on descriptive, rather than inferential, statistics. As a result, the impact of sampling error is typically not accounted for, and estimates do not provide formal measures of uncertainty. Last, as we discuss below, existing inferential methods used for estimating rates of dispersal and arrival times have their own limitations, which become particularly problematic when dealing with narrower geographical and chronological spans as in this case. Because of these limitations in data and methods, reliable estimates on whether, when, and where we observe variations in the rate of rice farming dispersal are currently not available, hindering our ability to postulate more robust hypotheses on its nature.

### Inferring dispersal rates and arrival time

Dispersal rates of farming are typically estimated from spatiotemporal analyses of either direct (e.g., radiocarbon dates on charred macrofossil remains, charcoal, bone collagen, etc.) or indirect (e.g., material culture) lines of evidence. While there is a substantial body of literature spanning nearly four decades, with very few exceptions (*12*), the analytical framework has predominantly focused on attempts to refine the estimate of the arrival dates via Bayesian phase models (*15*, *36*, *37*) or on inferring the speed of crop dispersal via regression-based analyses (*4*, *7*–*9*, *38*, *39*).

The application of Bayesian phase models for regional studies was originally introduced to study Late Glacial human occupation in Northwest Europe (*40*) and has since been used to study a variety of similar phenomena [e.g., (*41*) for a recent application]. The approach is effectively an adaptation of models typically designed to investigate the stratigraphic chronology of individual sites and has the benefit of providing estimates of arrival dates while taking into account the uncertainties associated with sampling and measurement errors. Leipe and colleagues (*15*) have recently adopted this approach to investigate the dispersal of rice farming in eastern Japan. Their work confirmed the earlier chronology of northern Tohoku compared to southern Tohoku and even led to the suggestion of an earlier uptake of farming in the Chubu region before northern Kyushu [although this latter result was entirely dependent on one questionable outlier date; see (*35*)].

The analyses by Leipe and colleagues (*15*), however, highlight two current limitations of this work. First, regional Bayesian phase models depend on how spatial units (i.e., “regions”) are being defined. Larger regions would provide more samples and lower uncertainty in the estimated parameters but at the expense of potentially missing important internal variations in arrival times. For example, Leipe and colleagues (*15*) suggest a starting date of rice farming in the Kanto region around the start of the sixth century BC, several centuries before the chronology suggested by the previous authors [cf. (*24*)] and an earlier date compared to their estimate for northern Tohoku (ca. third century BC). These figures either indicate that the region with the greatest resistance to the dispersal of farming was indeed southern Tohoku or that such a boundary was located somewhere within Kanto [as suggested by other authors, e.g., (*26*)]. These differences of a few hundred kilometers can have profound implications in examining different hypotheses on why the dispersal of rice agriculture slowed down.

The second limitation of regional Bayesian phase models is the sample interdependence, particularly when some sites contribute a disproportionately higher number of dates within a region. Ignoring this issue is effectively the same as considering a sample of 20 dates from 20 sites for a given region to be equivalent to a sample of 20 dates from a single site from that same region. The former would be more representative of the regional arrival date for agriculture, while the latter effectively provides an estimate of the arrival date to that particular site. While this is an extreme example, ignoring sample interdependence can lead to biased estimates (see fig. S16 and section S3.1 for a demonstration with a simulated dataset). The problem can be solved by either reducing the data to the earliest dated sample from each site or using a more complex model that accounts for the hierarchical structure of the data.

In contrast to Bayesian phase models, regression-based analyses are typically used with the objective of estimating the speed of the diffusion process rather than an accurate estimate of arrival dates. These analyses consist of fitting to the radiocarbon dates the geographic distance between sampling locations and a putative point of origin of the dispersal process, and the estimated slope is then used to obtain the diffusion rate. The approach has seen a number of additional features, including the use of alternative distance metrics, formal comparisons of putative points of origin, and spatially explicit models that account for geographic variation in the dispersal process (*4*, *8*, *38*, *39*). Despite differences in methodology (particularly with regard to how error ranges are calculated and reported), this wealth of case studies provides some benchmark estimates on the speed of the dispersal process, with figures around 0.6 to 1.3 km/year for Europe (*9*, *10*, *14*), 2.4 ± 1.0 km/year for South Africa (*42*), and values from 0.45 to 2.88 km/year for different parts of tropical South America (*6*).

While the possibility of direct comparison of dispersal rates makes these regression models highly appealing, particularly from the standpoint of reconstructing the generative process behind these patterns, there are several methodological issues related to the application of these methods. First, in contrast to Bayesian phase models described above, these regression analyses commonly ignore measurement errors associated with individual dates and fit models using median calibrated dates. As noted by Riris and Silva (*43*), this approach effectively dismisses the uncertainty associated with individual dates, and hence, in the best-case scenario, error estimates of the dispersal rates will be too low, and in the worst case, the estimate itself can be biased, particularly when the time range of analyses includes plateaus in the calibration curve as is the case with the first half of the Yayoi period (see fig. S1 and section S1).

The second issue stems from how samples are selected for analyses to capture the earliest dates associated with farming. While theoretically justifiable, the practical decision is clearly dependent on the spatial scale of analyses (i.e., “earliest” where?). Many earlier works have not provided the exact filtering protocol, although more recent works (*16*, *44*) offer more explicit and reproducible criteria. A more practical solution consists of fitting a quantile regression model, where the relationship between the predictors and the dependent variable is based on specific, user-defined percentiles. Several authors (*38*, *39*, *43*) have used this approach to model the earliest date using the full sample available. Regression-based methods can also be adapted to investigate possible variations in the dispersal rate, using techniques such as nonlinear regression (*39*), geographically weighted regression (*8*), or geostatistical interpolation (*4*, *14*). These solutions can reveal key variations in dispersal rates, which, in turn, are interpreted as evidence of low versus high receptivity of farming practices or demic versus cultural diffusion processes. However, it is hard to discern whether observed variations in the estimated rates of dispersal are genuine or just the consequences of calibration and measurement error or variation in sample structure.

Here, we use a Bayesian hierarchical Gaussian process quantile regression (GPQR), which combines the principles of Bayesian phase model and quantile regression while accounting for the full uncertainty of each date, sampling independence, and spatial variation in dispersal rates (see Materials and Methods and section S2). Our inferential method provides both global and local estimates of the rate of dispersal, with the latter representing the average local cumulative speed from the putative point of origin (in this case, Northern Kyushu). To maximize the reliability of our dated samples and avoid issues regarding old wood and reservoir effects or questionable stratigraphic associations of short-lived dates, we considered only direct ^14^C dates on charred rice grains (Fig. 1). We account for the measurement error of individual radiocarbon dates using the same modeling protocol commonly used in the Bayesian analyses of radiocarbon dates. We used quantile regression with the 90th and 99th percentiles to specifically look at the distribution of the earliest local arrival dates and a Gaussian process model to account for variation in the local dispersal rate.

Arrival dates were estimated using a hierarchical Bayesian phase model, which accounts for the problem of sampling independence discussed above (see Materials and Methods and section S3). Spatial units were defined by aggregating prefectures with sites yielding similar local dispersal rates from the GPQR model, ensuring a geographic subdivision that can highlight better differences in arrival dates between, rather than within, areas. We considered an unconstrained model (model *a*) where the arrival date of each area was solely determined by the radiocarbon dates of the focal area and a partially constrained model (model *b*) assuming a wave of advance dispersal in western Japan. Model *a* effectively allows for any possible routes of dispersal but yields estimates with higher uncertainties. In contrast, model *b* offers lower levels of uncertainties by imposing an origin of rice farming in northern Kyushu and a wave-of-advance diffusion for western Japan that is aligned with the current consensus in the literature.

## RESULTS

Average dispersal rates obtained from the GPQR ranged between 0.9 and 2.38 km/year [90% HPDI (highest posterior density interval)], with negligible variation between the 90th and 99th percentile models (figs. S14 and S15 and tables S1 and S2). Median posterior estimates (1.42 km/year for the 99th percentile and 1.33 for the 99th percentile model) were slightly higher than the average rate of agricultural diffusion observed in Europe (*9*, *10*), and the difference is even stronger in the case of a nonspatial version of the model, which yielded a median estimate of 2.00 km/year (90% HPDI interval of 1.59 to 2.51 km/year; fig. S3). The difference between the spatial and nonspatial model was most likely determined by the fact that the quantile regression is designed to capture the distribution of extreme observations (in this case, the earliest dates) and hence biased toward the fastest estimates for a given distance. In the GPQR, this is accounted for as a local deviation, while in the nonspatial regression, this is part of the global model.

**Fig. 2.**
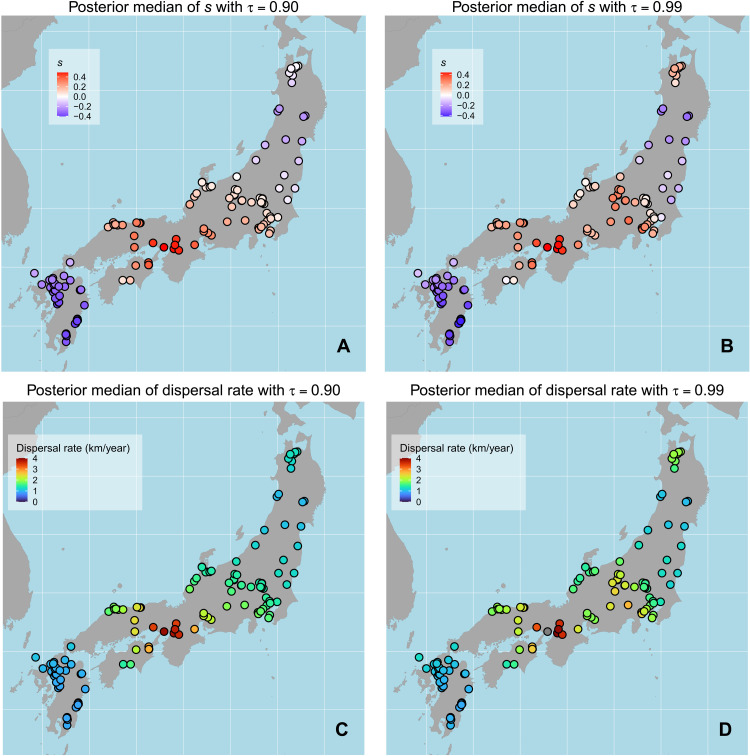
Local median posterior estimates of GPQR parameters. Deviations from the average slope parameter for the 90th percentile (**A**) and 99th percentile (**B**) models (positive values: faster dispersal rates; negative values: slower dispersal rates). Local rates of dispersal (in kilometers per year) for the 90th percentile (**C**) and 99th percentile (**D**) models.

Our analyses revealed substantial geographic variation in the dispersal rate (see Fig. 2), comparable to those observed elsewhere on a continental scale. Estimates of the length parameter ρ (fig. S14; see section S2 and figs. S4 and S5 for interpretation) indicate fairly wide regions (~300 km) yielding similar dispersal rates, with median estimates as high as 4 km/year on one end and below 1 km/year on the other. The model suggests that the rate of dispersal was initially slow within the island of Kyushu (below 1 km/year), accelerated in Chugoku and especially in Kansai (up to 4 km/year), and decreased its speed but maintained an above-average rate in Chubu (ca. 2 km/year), before slowing down from Kanto northward (1 km/year or less). The exception within this general pattern can be captured in the 99th percentile model, where the northernmost region of Honshu Island is associated with higher dispersal rates (ca. 2 km/year) compared to the rest of eastern Japan, providing support for the presence of a leapfrog transmission to this area.

**Fig. 3.**
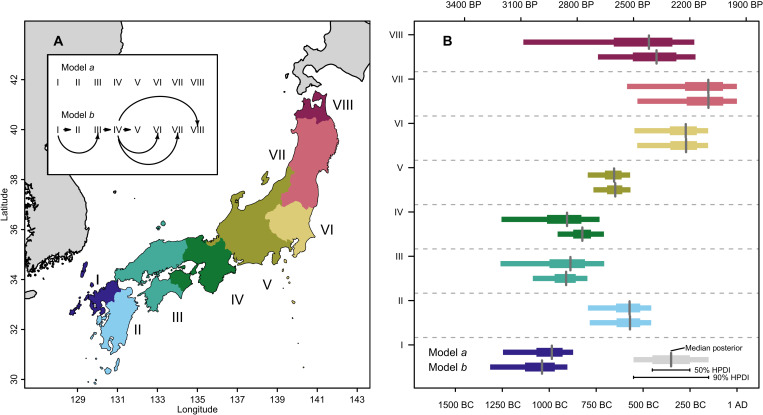
Estimated arrival dates of rice in different areas. Geographic areas used for the analyses and the constraint relationships defined in the two hierarchical models (**A**). Posterior distribution of ν*_k_* (i.e., arrival date) in the eight areas for each of the two models (**B**).

Estimates of the arrival time in the different areas obtained from the Bayesian hierarchical model are aligned with these findings (Fig. 3; see also figs. S18 and S19 and table S4). In the unconstrained model (model *a*), the estimated arrival date of rice farming in northern Kyushu (Area I) is between 1176 and 845 BC (90% HPDI; see table S5), confirming this area to be that with the earliest adoption of rice farming in Japan [contra (*15*); see fig. S20]. The next two earliest areas, comprising Chugoku, Shikoku, and Kansai, have estimated arrival dates between 1061 and 779 BC for Area III (90% HPDI) and between 946 and 703 BC for Area IV (90% HPDI) in the constrained models and with wider posterior density intervals in the unconstrained models. Arrival dates for Area II (central and southern Kyushu) and Area V (Chubu region) are very similar, the former yielding estimates between 735 and 430 BC (90% HPDI) and the latter between 754 and 560 BC (90% HPDI) in the constrained model. The largest chronological gap between geographically adjacent regions is recorded northeast of Area V, the same regions where we start to observe below-average local rates of dispersal in the GPQR model. Estimates of arrival time in Area VI (most of Kanto region, excluding Kanagawa prefecture) are between 471 and 124 BC, ca. 375 years (90% HPDI: 133 to 557 years; fig. S21) after the arrival in Area V, while Area VII yielded the latest median arrival date (152 BC, 90% HPDI: 434 BC to 42 AD) in the constrained model. As for the results of the GPQR with the 99th percentile, the phase model does confirm that rice cultivation arrived in northern Tohoku (Area VIII) before neighboring regions (Areas VI and VII), with an estimated arrival time between 709 and 203 BC (90% HPDI) in the constrained model. While the comparatively smaller sample size for this region has led to fairly large posterior intervals, the unconstrained model nonetheless provides support for a leapfrog transmission in northern Tohoku, with the probability of rice arriving in Areas VI and VII before VIII being 0.19 and 0.12, respectively (fig. S20).

## DISCUSSION

The introduction and dispersal of rice farming in the Japanese islands are a defining moment that sets the foundations of the subsequent genetic, linguistic, and cultural variations in the archipelago (*21*, *45*, *46*). Our analyses have confirmed that this process was characterized by substantial regional variation in its pace, with local episodes of slowdowns and accelerations in the dispersal of rice farming.

We have strategically traded off quantity for quality, focusing only on direct dates from charred rice remains, and used novel Bayesian inferential tools capable of handling different sources of uncertainties in our dataset, addressing many of the issues affecting previous studies aiming to estimate the rates of dispersal and arrival times. We demonstrated that inferring front speeds while ignoring the uncertainty associated with individual dates can drastically change estimates, particularly in the presence of calibration plateaus (see figs. S1 and S2) and that not accounting for sample independence can affect the conclusion of phase models applied in regional settings. The methods that we introduce were tested with simulated data (see sections S2.2 and S3.1) and are particularly well suited for handling smaller geographical and temporal windows of analyses that are typically characterized by smaller sample sizes and weaker signals. Furthermore, the hierarchical version of the Bayesian phase model provides a solution to the uneven sampling intensity that often characterizes legacy datasets. Our Gaussian process model offers an alternative to simple geostatistical interpolations of arrival dates used in previous studies. It provides a robust method for detecting changes in dispersal rates that accounts for variations in sample size via partial pooling, i.e., estimates for areas with fewer samples are based partially on neighboring regions with a more abundant number of radiocarbon dates. Although the relative density of dates on short-lived samples is higher compared to studies with continental scales of analyses, our limited sample size led to fairly larger posterior estimates. This is, however, in part due to the fact that our analyses fully account for the measurement errors associated with individual dates in contrast to previous studies based on median calibrated dates. Both the approaches that we introduce can be augmented by including independent variables and, hence, can offer a foundation for directly testing more specific dispersal hypotheses.

In general terms, our estimates point to earlier arrival dates for all regions, as, in contrast to most existing studies, we base our figures on formal inference and not on the visual inspection of sample dates. We estimate that the introduction of rice agriculture in northern Kyushu occurred around the turn of the first millennium BC. Evidence for rice farming occurring in the Japanese islands during this time or earlier is patchy and often controversial, although their density is higher in northern Kyushu compared to that in other parts of the archipelago [cf. figure 83 in (*29*)]. Several studies have shown impressions of rice grains on potsherds predating the Initial Yayoi period (*35*). Nakazawa and Ushino (*47*) identified a rice grain impression from a ceramic vessel recovered at Itaya III site in Shimane prefecture (Fig. 1) attributed to the Maeike phase of the Final Jomon period. However, its typological classification is controversial [see (*48*) for suggestions of a later chronology], and the lack of direct dating and a reliable absolute chronology of the Maeike phase do not provide sufficiently robust evidence for farming occurring earlier in the Chugoku region. Leipe and colleagues (*15*) argue for the possibility of an earlier adoption of rice farming in the Chubu highlands based on the direct date of a single rice grain (IAAA-83092, 2889 ± 29 ^14^C BP) from the Chikaraishijori site in Chubu (Fig. 1). However, the sample is a clear outlier when compared to the other charred rice dates from the same site, and one of the authors acknowledges in a subsequent study that its identity could not be verified (*35*). Removing this sample provides a much later date for this region, and our model (fig. S20) strongly indicates that the arrival of rice in northern Kyushu (Area I) predates the arrival in the Chubu Highlands (Area V).

The expansion of agriculture outside northern Kyushu was characterized by major differences in the eastward and southward dispersal. The eastward expansion within the cultural area of preexisting Tottaimon pottery (Areas III and IV) was fast paced, reaching dispersal rates of more than 4 km/year, while the adoption of farming in southern Kyushu was significantly slower with later arrival dates compared to Chugoku, Shikoku, and Kinki regions (Fig. 2 and fig. S20). The fast dispersal rate in these regions has been pointed out by several authors in the past [e.g., (*24*)], although our chronology is substantially earlier, particularly for Kinki (Area IV). The slower dispersal rate in southern Kyushu has been hinted at in the past, with authors suggesting that the underlying cause was due to rice farming being less suited to local topographic and soil conditions (*49*, *50*). Evidence of earlier farming sites in areas south of northern Kyushu has been advocated, but most are based on indirect lines of evidence, and a reliable chronological assessment is still lacking (*29*, *51*).

The expansion of rice farming in central and eastern Japan is characterized by a slower pace compared to that seen for Areas III and IV, albeit with considerable variability. The adoption of early wet rice farming in central Japan is noteworthy, mostly due to the lack of paddy field sites dated to this period in the area, which has led some authors to suggest the presence of dry rice farming [e.g., (*26*) for Nakayashiki site in Kanagawa; Fig. 1; but see (*52*) for an alternative interpretation]. While the pace of dispersal is slower than in Kinki, the presence of hybrid pottery styles (i.e., Joukonmon pottery) does hint at the presence of different forms of cultural interaction (*53*), although our analyses suggest that the front speed in this area was faster than previously thought. Kobayashi (*26*) identifies two “Jomon Walls” in the area, one located near the waist of Japan (*53*), approximately between Areas IV and V (the “Chubu Wall”), and one located in the Kanto region (the “Tone River Wall,” between Areas V and VI). The former “wall” corresponds to the expansion limit of the new Ongagawa pottery (*33*) and has been considered by many as the point of the largest slowdown in Honshu. Our analyses confirm a slower rate of dispersal in eastern Japan compared to most of western Japan, but we identify that the largest discrepancy in arrival dates appears to be between Areas V and VI (Fig. 3 and fig. S21).

The expansion of rice farming beyond Area V is further characterized by higher levels of uncertainty, particularly due to the smaller sample sizes in the Tohoku region. Our analyses do confirm that rice farming was present in the Tsugaru plain in Aomori prefecture (Area VIII) before other regions in eastern Japan (Areas VI and VII; fig. S20). However, we found no evidence supporting a coastal route via the Sea of Japan followed by a possible downward expansion via the Pacific coast as claimed by some authors (*24*), based on the presumed movement of material culture (*54*). Instead, our analyses identified Area VII (Tohoku excluding Aomori) to be the last region of those analyzed to witness the arrival of rice farming (although with considerable uncertainty; see fig. S20).

Several putative factors could explain the dispersal rates we inferred. Although not part of our analyses, the colder climate in Hokkaido most likely hindered the dispersal of rice farming in the north (*31*), while the lack of suitable terrains might have played a role in the slowdown of its expansion into southern Kyushu (*49*, *50*). However, the role played by environmental factors is less clear for the rest of the archipelago. The cooler climate of Tohoku could have been less suitable for the cultivation of rice, while the mountainous regions of central Japan might have acted as a transmission barrier. Our analyses have confirmed an early uptake of farming in Aomori prefecture (Area VIII), where several early paddy field sites, such as Sunazawa and Tareyanagi, have been identified. These sites were, however, abandoned after just a few centuries, and the local communities reverted to a predominantly hunting and gathering economy, suggesting that rice farming was not a fully consolidated part of their subsistence economy. Takase (*55*) suggests that these abandonments were triggered by a local flood event, but the lack of subsequent recovery of rice agriculture hints at the possibility that northern Tohoku was indeed at the edge of the thermal niche for rice cultivation. Previous studies (*26*, *46*) have suggested that the location of the largest slowdown in the dispersal of farming occurred at the so-called “waist of Honshu” (between areas IV and V in our study), with the mountainous area in Chubu effectively acting as a topographic barrier. While we do indeed observe a delay of a few hundred years in this region (fig. S21), our analyses suggest that a larger slowdown took place between areas V and VI. The lack of any prominent variation in topography, soil, and climate within these regions east of the Chubu mountains suggests that other processes might have played a greater role in determining the rate of dispersal here.

Discussions regarding the relative contribution of demic versus cultural diffusion have been comparatively limited in Japanese archaeology, although some [e.g., (*56*)] have suggested that the faster dispersal rate in Western Japan was more likely associated with migratory movements, while the slower dispersal in Eastern Japan is suggestive of a greater role played by cultural transmission. This is, in principle, in line with the expectations suggested by other studies outside of Japan (*5*), but it is worth noting that a pure cultural diffusion (i.e., an intergroup transmission without genetic admixture) was extremely unlikely, given the complex knowledge such as water management and transplanting required in rice cultivation. While some authors have argued for a difference in the receptivity of rice cultivation among western and eastern Jomon hunter-gatherers (*57*), it is worth considering whether a full change in the subsistence economy can be triggered by intergroup transmission alone. A possible alternative scenario is a mixed demic-cultural diffusion, where the dispersal of rice farming was promoted via intermarriages and group fission events. The intermarriage between farming groups and hunter-gatherers might have been conditioned by preexisting social networks, and hence, preexisting cultural boundaries might have hindered the dispersal of rice farming. At the turn of the first millennium BC, the Japanese archipelago was characterized by three major ceramic zones [Tottaimon in the west, Fusenmon in the center, and Kamegaoka in the northeast; (*18*, *33*)] and higher population densities in eastern Japan compared to the west (*32*). While the degree by which the three ceramic zones represent cohesive social networks is speculative, it is worth considering the possibility that their presence might have conditioned the rate of dispersal of farming. In western Japan, expanding migrant communities had potentially less competition for space and, at the same time, might have become integrated into preexisting social and intermarriage networks of the Tottaimon zone. The slowdown in central Japan could then be attributed to the consequence of crossing a cultural boundary that brought expanding agriculturalists into contact with communities outside this social network, along with a necessary adaptation to the different topographic settings of central Japan, characterized by smaller coastal plains and narrower fluvial valleys. Similarly, the dispersal into northeastern Japan entailed a further transition into the Kamegaoka zone, which might have again contributed to a slowdown in the diffusion of rice farming rate into this region.

The picture currently emerging from our analyses reveals that even within the relatively confined space of the Japanese islands, the dispersal of farming was characterized by a substantial degree of heterogeneity. While variations in the rate of dispersal have often been approached simplistically by the contrast of demic versus cultural diffusion, we argue that the interplay of different environmental settings, the density of incumbent communities of hunter-gatherers, and preexisting networks of social connectivity are plausible alternative explanations that are worth pursuing here and elsewhere. The Bayesian methods introduced here were primarily focused on detecting more accurately where and when we can observe variations in the dispersal of rice agriculture, but at its core, it provides the necessary framework required to evaluate these hypotheses in the future.

## MATERIALS AND METHODS

### Radiocarbon data

We compiled a ^14^C dataset of 439 charred samples of direct dates on *Oryza sativa* grains from 218 archaeological sites located in the Japanese islands. Dates were collected from site reports, journal articles, and the ^14^C database of the National Museum of Japanese History (*58*). We filtered these data by excluding samples from the Ryukyu Islands and Hokkaido, samples yielding uncalibrated ^14^C ages smaller than 1000 BP, and those with possible contamination of dated carbon or without a reliable taxonomic identification. The resulting dataset (data file S1) consisted of 294 dates from 132 site locations.

### Gaussian process quantile regression

We modeled the geographic variation in the rate of dispersal from a putative origin point located at Ukikunden Shell Midden in Northern Kyushu, where the earliest charred rice date in our dataset was recovered, using a Bayesian hierarchical GPQR defined as follows

$$\theta_{i}\sim \text{AsymLaplace}(\mu_{i},\lambda ,\tau )$$

$$X_{i}\sim \text{Normal}\;(f(\theta_{i}),\sigma_{i})$$

where θ*_i_* is the true calendar date of the earliest charred sample identified at each site *i*, *f*(𝜃_i_) is its corresponding ^14^C age on the IntCal20 calibration curve (*59*), *X_i_* is the observed conventional radiocarbon age of the sample, and σ*_i_* is the square root of the sum of the squares of the samples’ ^14^C age error and the error on the calibration curve. The core of the model is the asymmetric Laplace likelihood (*60*), where τ is the quantile of interest, λ is the scale, and m*_i_* is the location parameter defined by the linear expression

$$\mu_{i}=\beta_{0}+d_{i}(\beta_{1}-s_{i})$$

where b_0_ is the intercept, *d_i_* is the great-arc distance (in kilometers) between the focal site *i* and the putative origin point, b_1_ is the average negative reciprocal of the dispersal rate, and *s_i_* is a spatially autocorrelated random effect representing the local deviation of the dispersal rate. More specifically, *s_i_* is modeled as a multivariate normal distribution

$$s_{i}\sim \text{MVNormal}(0,\Sigma )$$

with a vector of mean equal to 0 and the covariance matrix defined by a quadratic exponential model

$$\sum_{i,j}=\eta^{2}\text{exp}(-0.5{\left(D_{i,j}/\rho \right)}^{2})+I_{i,j}\varepsilon^{2}$$

where the covariance Σ_*i*,*j*_ between pairs of sites *i* and *j* declines exponentially as a function of their great-arc distance *D*_*i*,*j*_ at a rate defined by the length-scale parameter ρ, and with a maximum covariance equal to the square of the marginal deviation η. The term *I*_*i*,*j*_ ε^2^ provides additional covariance ε^2^ in case the *i* and *j* are identical (i.e., *I*_*i*,*j*_ is an identity matrix equal to 0 when *i* ≠ *j* and 1 when *i = j*). In practical terms, because there is only one local deviation per site, this part of the equation is not relevant. However, with small values of ρ and *D*_*i*,*j*_, setting ε^2^ to 0 can be problematic for algorithmic reasons (nonpositive eigenvectors), and hence, a small constant of 10^−6^ was assigned to this parameter. We also considered a nonspatial version of the same model where m*_i_* was simply defined by a linear equation without a random effect, i.e., b_0_ + *d_i_*b_1_ (see section S1.1).

We used the weakly informative priors informed by prior predictive checks and realistic ranges of dispersal rates inferred from other studies (see section S2.1 and figs. S7 and S8). To establish the robustness of the proposed approach, we generated a simulated dataset with a comparable sample size to our observed data and fitted our GPQR model (see section S2.2 and figs. S9 to S11). Results indicate a good performance with all fixed parameters and most random effect parameters within the 95% higher posterior density interval.

Following previous studies, we fitted our models using the 90th and the 99th percentiles (i.e., τ = 0.9 and τ = 0.99). The latter represents more closely the earliest arrival date of rice, but it is more susceptible to potential outlier dates.

### Hierarchical phase model

We estimated the arrival date for eight geographic areas using a Bayesian hierarchical phase model (see section S3). The spatial extent of the areas was defined on the basis of a combination of prior archaeological knowledge while ensuring that the dispersal rates estimated by the GPQR were internally homogeneous. The eight areas are as follows: I (Fukuoka, Saga, and Nagasaki prefectures), II (Oita, Miyazaki, Kagoshima, and Kumamoto prefectures), III (Chugoku region, Ehime, and Kochi prefectures), IV (Kansai region, Kagawa, and Tokushima prefectures), V (Chubu region and Kanagawa prefecture), VI (Saitama, Tokyo, Chiba, Gunma, Tochigi, and Ibaraki prefectures), VII (Fukushima, Yamagata, Miyagi, Iwate, and Akita prefectures), and VIII (Aomori) (table S3). The separation of the island of Kyushu between Areas I and II was based on the distinct nature of the earliest farming communities of northern Kyushu, while eastern Shikoku was assigned to Area IV rather than III based on the result of the GPQR that suggested a faster dispersal rate in those areas closer to sites in eastern Kansai. Kanagawa had several early sites and faster dispersal rates than the rest of Kanto and hence was assigned to Area V, and lastly, Aomori was kept separated from the rest of Tohoku (Area VII) to evaluate the leapfrogging hypotheses based on the presence of earlier paddy fields in the Hirosaki plain area.

In contrast to typical regional phase models where sample independence is either ignored (i.e., multiple dates from the same site represented within each phase) or controlled by limiting the number of specimens to a unit per site, we first modeled the distribution of all charred rice dates within each site *i* and estimated its start date a*_i_* as follows

$$\theta_{i,j}\sim \text{Uniform}(\alpha_{i},\alpha_{i}+\delta_{i})$$

$$X_{i,j}\sim \text{Normal}\;(f(\theta_{i,j}),\sigma_{i,j})$$

where θ_*i*,*j*_ is the calendar date of the sample *j* from the site *i*, and δ*_i_* is the duration of the rice use at the focal site. The measurement error of the θ_*i*,*j*_ was modeled using the same procedure used in the GPQR model. The distribution of start parameters a*_i_* within a given Area *k* was modeled using a uniform probability distribution with start and end dates ν*_k_* and υ*_k_*, where the former is our primary parameter of interest representing the arrival of rice in the focal region. As for the GPQR model above, we tested the robustness of our model on simulated data (see section S3.1).

We considered two different models based on assumptions (or lack thereof) on the relationship of ν*_k_* for the eight areas (see Fig. 3, left). In model *a*, we assumed no constraint, and hence, estimates of ν*_k_* were effectively made independently for each area. In model *b*, we instead assumed a wave of advance dispersal between northern Kyushu (Area I) and Kansai (Area IV), hence imposing the constraints ν_I_ > ν_II_ and ν_I_ > ν_III_ > ν_IV_, so that Area I was earlier than Area II, Area III was earlier than Area IV, etc. Areas in central and eastern Japan (Areas V to VIII) were assumed to be later than Area IV, but we did not impose any constraints between them (i.e., ν_IV_ > ν_V_, ν_IV_ > ν_VI_, ν_IV_ > ν_VII_, and ν_IV_ > ν_VII_) to allow for possible leapfrog dispersals as hypothesized by some authors [e.g., (*24*)]. We used flat priors bounded between 5000 and 500 cal BP for ν*_k_* and υ*_k_* and weakly informative prior for δ (see section S3.2) for both models.

### Parameter inference

Model fitting was carried out in R v.4.1.0 (R Core Team 2021), using the nimble v.0.12.1 (*61*, *62*) and the nimbleCarbon v.0.2.1 (*63*, *64*) R packages. We used a Metropolis-Hasting adaptive random walk sampler for all parameters except for b_1_ and *s_j_* in the GPQR model, which were inferred using an automated factor slice sampler to account for correlation between the parameters. We ran four chains for all models, using 2 million iterations for the GPQR and 6 million iterations for the hierarchical phase model. In both cases, we dedicated half the iterations for the burn-in and thinned our sample to reduce file sizes (every 300 steps in the phase model and 100 in the GPQR). Convergence of the chains was checked using the Gelman-Rubin diagnostic and visual inspection of the trace plots.

## Supplementary Materials

### This PDF file includes:

Supplementary Text S1 Figs. S1 to S21 Tables S1 to S4 References
