# Supplementary material for: Bayesian analyses of direct radiocarbon dates reveal geographic variations in the rate of rice farming dispersal in prehistoric Japan
Source: Sci Adv. 2022 Sep 21;8(38):eadc9171. doi: 10.1126/sciadv.adc9171 (PMC9491708; doi:10.1126/sciadv.adc9171)
Supplement: Supplementary file 1 — Supplementary Text S1 Figs. S1 to S21 Tables S1 to S4 References [file sciadv.adc9171_sm.pdf]

Science Advances
AAAS

Supplementary Materials for

Bayesian analyses of direct radiocarbon dates reveal geographic variations in the rate of rice farming dispersal in prehistoric Japan

Enrico R. Crema *et al.*

Corresponding author: Enrico R. Crema, erc62@cam.ac.uk

Sci. Adv. **8**, eadc9171 (2022)
DOI: 10.1126/sciadv.adc9171

The PDF file includes:

Supplementary Text S1
Figs. S1 to S21
Tables S1 to S4
References

Other Supplementary Material for this manuscript includes the following:

Data S1

Supplementary Text S1

1. Handling the Impact of Radiocarbon Measurement Error and Calibration Plateau

Estimates of farming dispersal rates are typically obtained by regressing the distance of focal sites from a putative point of origin against the mean or the median calibrated radiocarbon date of the earliest evidence of farming on those sites (*6,9,10,38,42*). The rate is then computed by taking the negative (in case time is represented by BP and positive values) reciprocal of the slope coefficient (but see (*9*) for fitting time against distance instead). Confidence intervals of the derived rate estimates do not, however, account for the impact of the measurement error of the radiocarbon dates, nor the fact that these errors are non-random due to calibration effects. Figure S1 illustrates the implication of this issue, showing a hypothetical dispersal of 2.85 km/year with four observation points in calendar time (black points) and their corresponding median calibrated dates (red points). The latter converge to nearly identical values due to the effect of the calibration plateau, leading to a slope approximately equal to 0, which in turn corresponds to an estimated instantaneous dispersal.

A possible solution, first introduced by Steele (*65*), and later adopted in other studies (e.g. (*6,43*)), consists of fitting the regression model to iteratively sampled calendar dates from each calibrated distribution and obtaining multiple estimates from which confidence intervals are derived. While this solution accounts for the uncertainty of each measurement, it holds a conservative stance where effectively each point is treated separately, without acknowledging that these uncertainties are correlated to each other. Furthermore, these approaches often consist of aggregating the estimates of individual iterations but not their associated errors (but see (*43*)); hence the resulting confidence intervals could potentially fail to properly account for the uncertainty derived from sampling error.

1.1 Bayesian Quantile Regression

An alternative approach is to fit a hierarchical measurement error model, following the same principles adopted in Bayesian phase models (*66*), and more recently in radiocarbon-based demographic inference (*64*). In this case, the regression model can be conceptualised as follow:

$$\theta_i \sim \text{Normal}(\mu_\text{i}, \varepsilon) \quad [1]$$
$$\mu_\text{i} = \beta_0 + \beta_1 d_i \quad [2]$$
$$\text{X}_\text{i} \sim \text{Normal}(f(\theta_\text{i}), \sigma_i) \quad [3]$$

where θ_i is the true calendar date of the date at each site focal site *i*, d_i is the distance of the site from the putative source of dispersal, $f(\theta_\text{i})$ is its corresponding ^{14}C age on the IntCal20 calibration curve (*59*), and X_i is the observed conventional radiocarbon age of the sample, and σ_i is the square root of the sum of the squares of the samples' ^{14}C age error and the error on the calibration curve. Equation [3] effectively handles the measurement error of θ and establishes its relationship to the observed ^{14}C age and its associated error. In practice, equations [1] and [2] can be replaced by any model and following recent works advocating the use of quantile regression (*38,39,43*) we use an asymmetric Laplace likelihood (*60*), so that θ is defined as follows:

$$\theta_i \sim \text{AsymLaplace}(\mu_\text{i}, \lambda, \tau) \quad [4]$$

where μ_i is the same linear model as equation [2] above, and λ is the scale parameter, and the τ is the user-defined quantile of interest. Because we are interested in the distribution of the earliest dates, previous applications of quantile regression for dispersal processes employed extreme values such as 90th (τ=0.9) and 99th percentiles (τ=0.99).

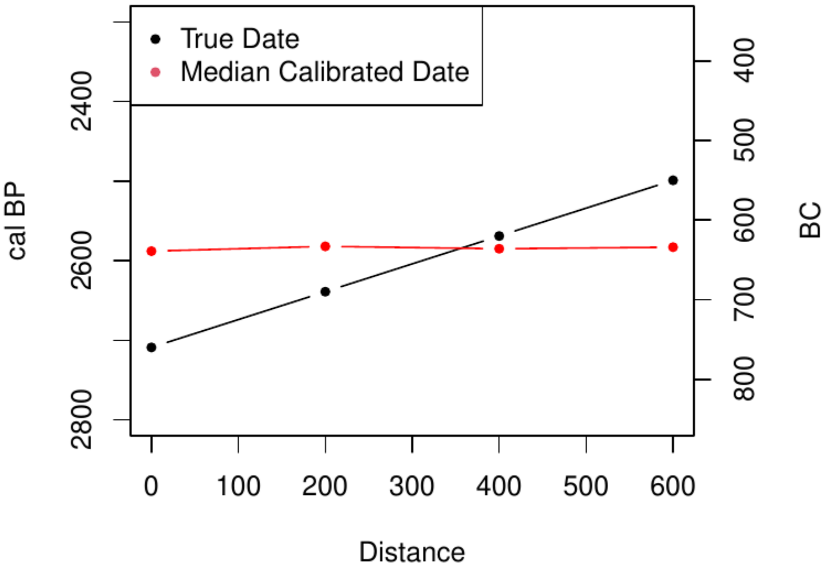

Fig.S1. Impact of systematic measurement error on a putative relationship between distance and time for dates falling the calibration plateau. True rate (black) shows a positive slope indicative of a dispersal rate of ca 2.85 km/year, whilst median calibrated dates (red) have a flat slope with an instantaneous (i.e. infinite) dispersal rate.

Figure S2 shows the result of the quantile regression model described above fitted to the earliest charred rice dates from each archaeological site, using as the putative source of dispersal the earliest date in our sample (Ukikunden Shell Midden in Northern Kyushu; 130.0087, 33.411, Fig.1 main text). We fitted our model using the following priors:

$$\beta_0 \sim \text{Normal}(3000, 200) \qquad [5]$$
$$\beta_1 \sim \text{Exponential}(1) \qquad [6]$$
$$\lambda \sim \text{Exponential}(0.01) \qquad [7]$$

setting τ equal to 0.9.

Posterior estimates of the model parameters were obtained using Markov Chain Monte-Carlo (MCMC) sampling via the *nimble* (*61*) and *nimbleCarbon* (*63,64*) packages in R (*67*). We used four chains, each with 6 million iterations (half dedicated to burn-in) and parameters sampled every 300 steps to reduce file size. We used Metropolis-Hasting adaptive random walk sampler for all parameters using default settings, with the exception of the age estimates θ, where we used an *adaptInterval* of 20,000 and an *adaptFactorExponent* to 0.1 which yielded better mixing in test runs. Convergence was evaluated using the Gelman-Rubin diagnostic and visual inspection of the trace plots. Results showed good mixing with the highest R-hat equal to 1.015..

Figure S2 contrasts this Bayesian model (shown in red) against a non-Bayesian quantile regression model fitted with the median calibrated dates using the *quantreg* (*68*) R package. The result mirrors the expectations of figure S1, with the median date model (in blue) showing a flatter slope and, consequently, a much higher expected rate of dispersal compared to the Bayesian model (in red). This discrepancy is due to the impact of the so-called 'Hallstatt plateau' in the calibration curve and is made evident by the larger discrepancies between the median calibrated dates and the median posterior values of θ. The discrepancy is almost absent for periods where the impact of the plateau is small and the model overall provides little additional information concerning individual dates. However, dates in the plateau region, which typically show wider and flatter posterior calibrated distributions, are largely "informed" by the overall model, with a resulting shrinking of the measurement error, and in this case, an offset of the resulting median date. While it is worth noting that this model does not account for regional variation in the dispersal rates, the reciprocal of the posterior of the parameter β_1 (figure S3) provides an estimate of the average dispersal rate of charred rice farming between 1.59 and 2.51 km/year.

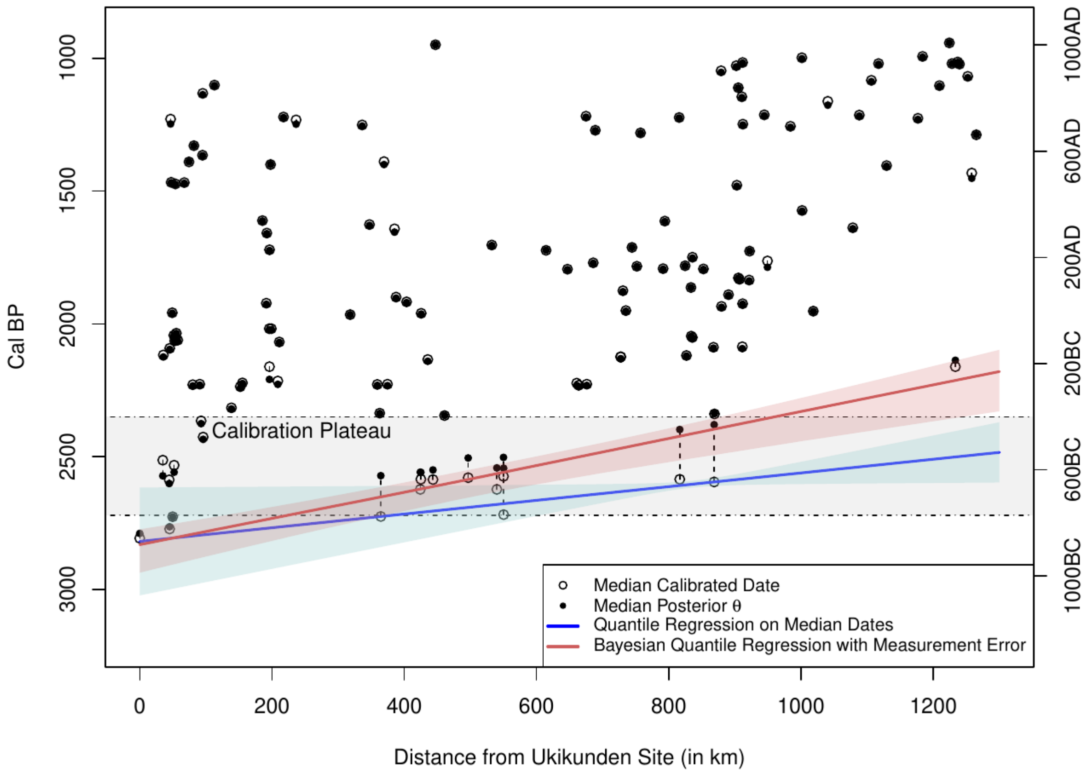

Fig.S2. Quantile regression model fitted on median calibrated dates (blue line) and accounting for measurement error via Bayesian hierarchical model (red line). Hollow dots are the median calibrated dates while the filled dots are the median posterior of the Bayesian model accounting for the overall model. The offset between median dates and the model posterior is largest in the calibration plateau region, where the calibrated dates generally show flat distributions and the overall model can provide the highest amount of additional information.

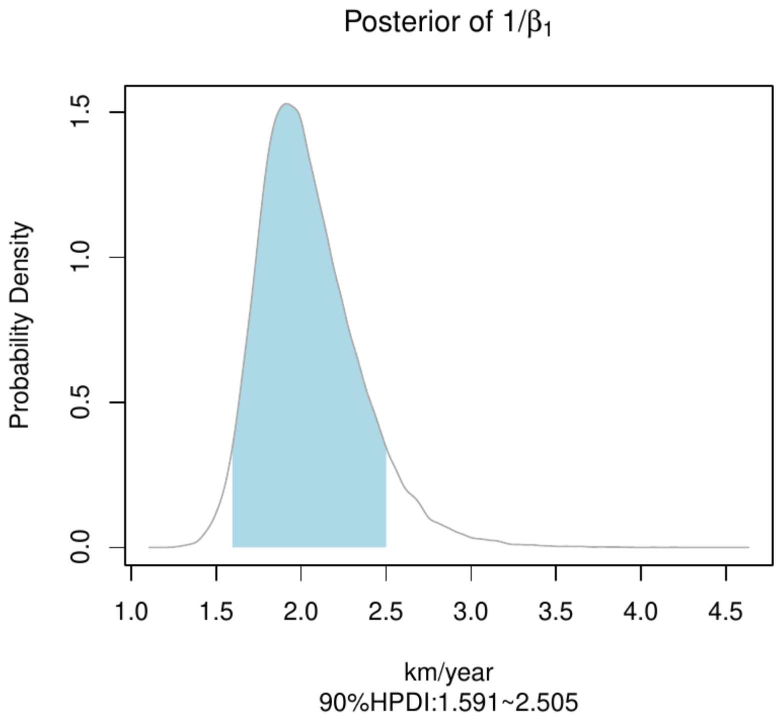

Fig. S3. Estimated dispersal rate for the spread of charred rice farming in Japan using Bayesian quantile regression.

2. Bayesian Gaussian Process Quantile Regression

The linear model formalised in equation [2] assumes that $1/\beta_1$ is constant across the geographic window of analyses and hence not able to identify local episodes of acceleration and slow-down in the dispersal process. A slight modification of the linear model can account for such local processes:

$$\mu_i = \beta_0 + d_i (s_i - \beta_1) \qquad [7]$$

Here the additional term s_i allows for a site-level variation in the dispersal rate. This local deviation parameter can be assumed to be a random effect within a hierarchical setting, but with the important additional assumption that s is spatially autocorrelated. A Gaussian process model (*69*) can satisfy these two assumptions so that the parameter is modelled as a multivariate normal distribution:

$$s_i \sim \text{MVNormal}(0, \boldsymbol{\Sigma}) \qquad [8]$$

with a mean of 0 and with a covariance matrix $\boldsymbol{\Sigma}$ defined by a quadratic exponential model (see also figure S4):

$$\boldsymbol{\Sigma}_{i,j} = \eta^2 \exp(-0.5(d_{i,j}/\rho)^2) + I_{i,j}\varepsilon^2 \qquad [9]$$

where the covariance $\boldsymbol{\Sigma}_{i,j}$ between sites i and j is portrayed as an exponential decay based on their inter-distance $d_{i,j}$ with length scale parameter ρ and maximum covariance equal to the square of the marginal deviation parameter η. The term $I_{i,j}\varepsilon^2$ provides an additional covariance ε^2 in case the i and j are identical.

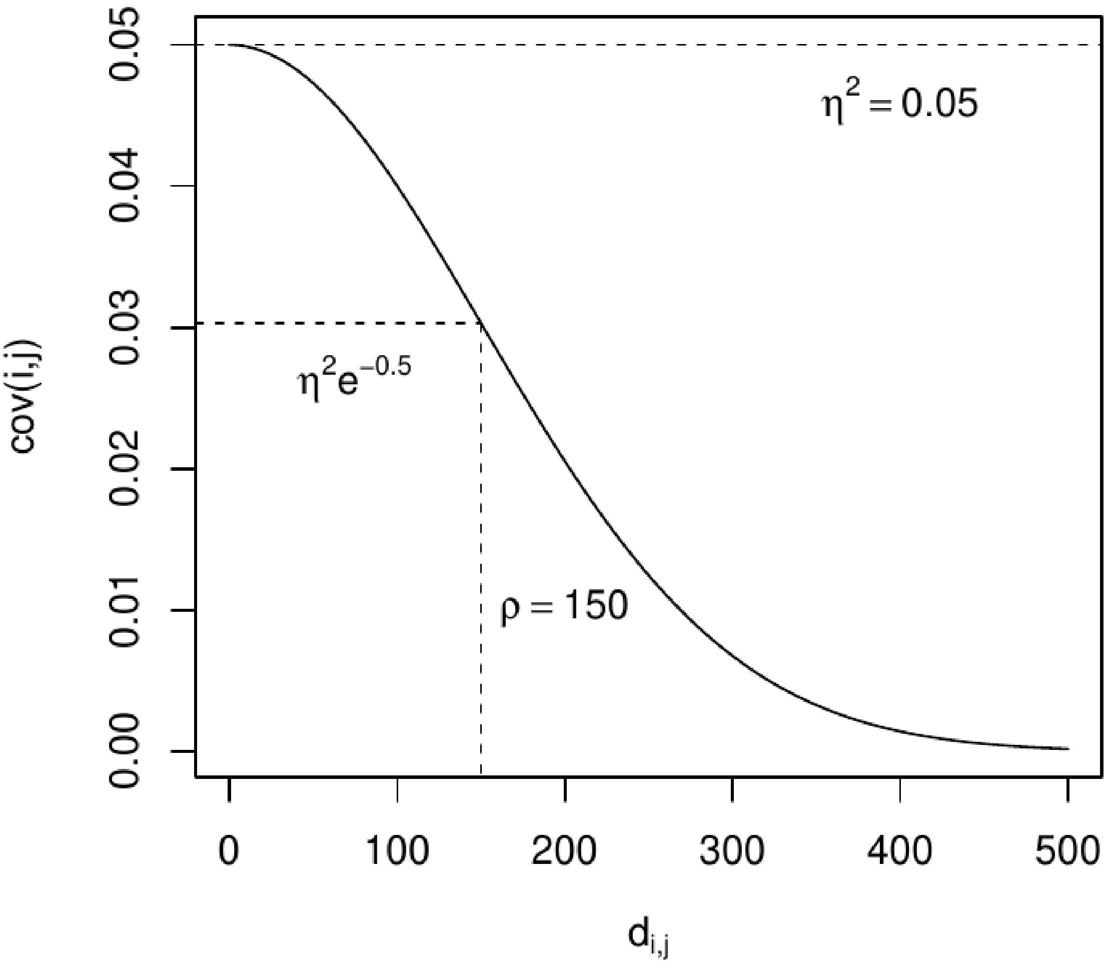

Fig.S4. The quadratic exponential model and its relationship to its parameters ρ, η, and $d_{i,j}$ assuming $i \neq j$.

Figure S5 shows the result of simulated dispersal rates under different settings of ρ and η. With all things equal, larger values of ρ yield wider regions with similar dispersal rates, whilst larger values of η lead to a wider range of values of *s*, and consequently greater diversity in the dispersal rate.

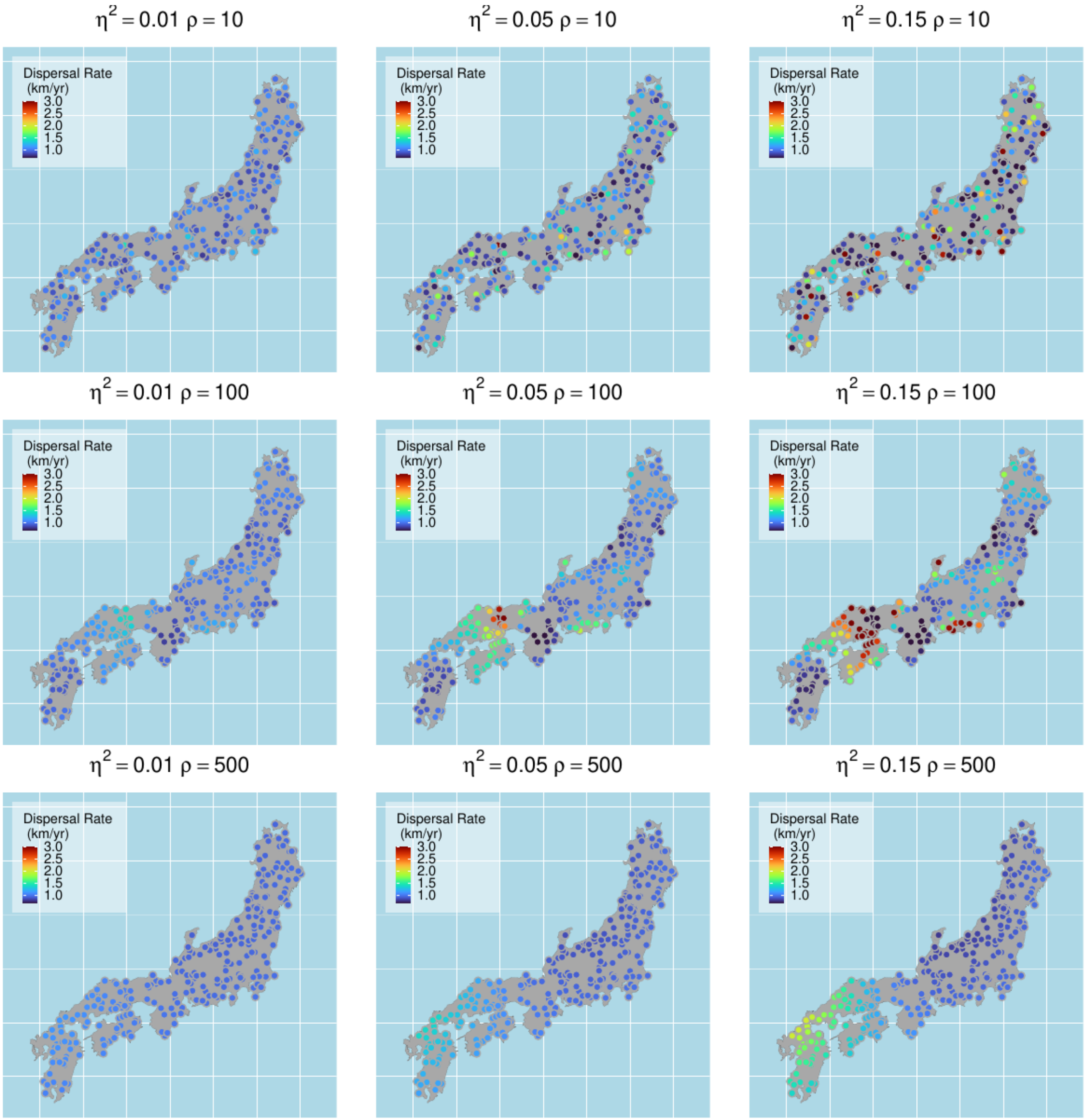

Fig.S5. Simulated local dispersal rates with different settings of ρ and η, with 300 site locations and $\beta_0 = 3000$ and $\beta_1 = 1$

It is worth mentioning here that the local deviation parameter s is based on the slope and hence should not be directly interpreted in terms of dispersal rate. For example with β_1 = -0.5 and s=0, the dispersal rate is equal to 2 km/year (i.e. -1/(0-0.5)), with s = 0.2 the rate increases to ca. 3.33km/year (i.e an increase of 1.33 km/year), whilst s = -0.2 decreases the rate to 1.42 km/year (i.e. a decrease of 0.58 km/year). In other words, the impact of s is symmetric towards the slope, but not towards the rate. This is due to the very nature of the reciprocal of slopes which accelerates quickly towards 0 with a limit of infinite velocity when the slope is equal to 0 (see figure S6).

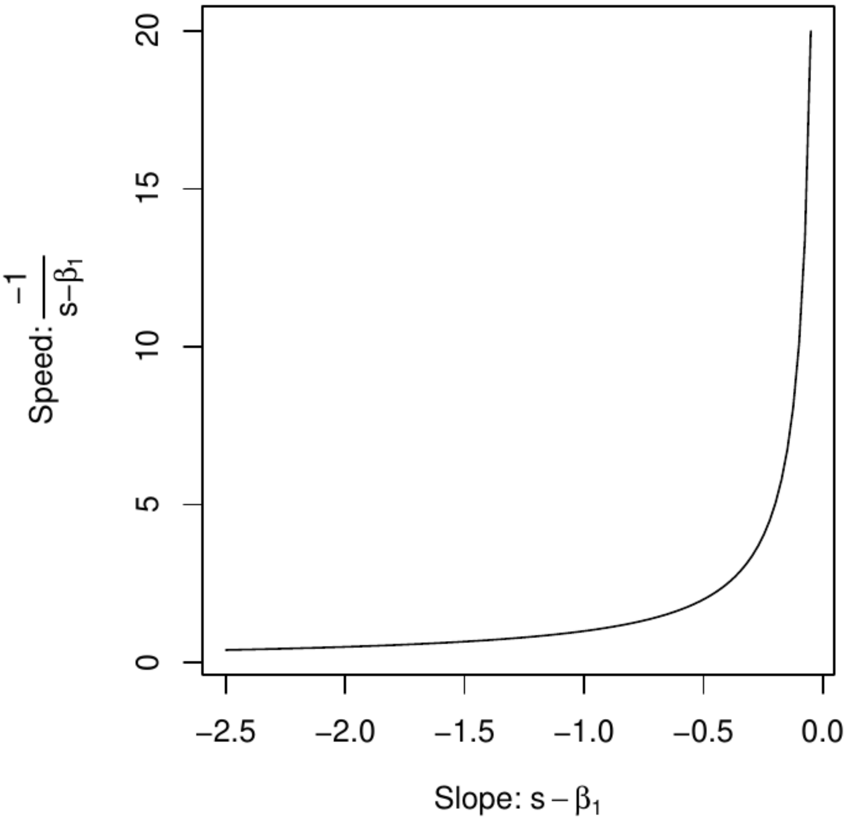

Fig.S6. Relationship between regression slope (here s-β_1) and its negative reciprocal, i.e. rate of dispersal.

2.1 Priors

Priors for the Gaussian Process Quantile Regression (hereafter GPQR) model were weakly informed by independent lines of archaeological evidence. As for the non-spatial quantile regression model described above, the intercept was based on a normal distribution with a mean of 3,000 and a standard deviation of 200 (see equation [5]) which broadly assumes the arrival of rice to be somewhere between the second half of the 2nd millennium BC and the first half of the 1st millennium BC. Sensible priors for the slope parameter can be based on known archaeological examples of farming dispersal rates, for our case this is equivalent to a reciprocal of s - β_1 . The variance of the local dispersal rate s is equal to η^2 in the case of a complete lack of spatial autocorrelation. This can be used as the basis for building a prior predictive check model to establish which prior combination yields sensible dispersal rates. Figure S7 shows the result when an exponential prior with a rate of 1 is applied for β_1 (cf. equation [6]) and an exponential prior with a rate of 20 is used for η^2. The results have a wide range of dispersal rates that include observed values from other archaeological contexts. Given the premise and assumptions of the spread of rice farming in Japan, the parameter was also censored to ensure that the dispersal rate is always positive.

The prior for the length scale parameter ρ was modelled using a gamma distribution modelled with a shape of 10 and a rate of 0.06 and truncated between 1 and 1350 km (the smallest and largest inter-distance between sampled sites). The gamma distribution enforces positive values, with a fairly long tail and, in this case, a mode around 200km. These settings ensure a fairly wide range of scales for regional patterns of local dispersal rates (Fig. S8).

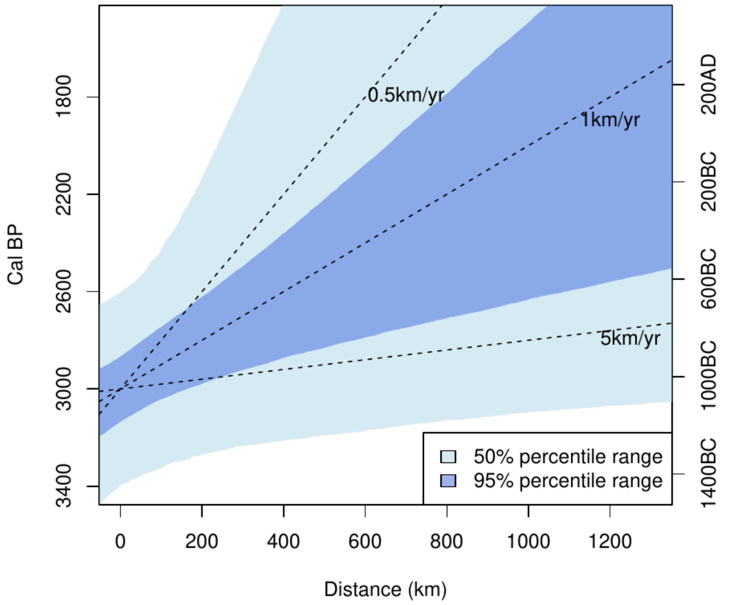

Fig.S7. Prior predictive check for β_0 (modelled as a Gaussian with a mean of 3000 and a standard deviation of 200), β_1 (modelled as an exponential with a rate of 1), and η^2 (modelled as an exponential with a rate of 20). The slope was derived by sampled values of s - β_1, with s drawn from a gaussian with a mean of 0 and a standard deviation equivalent to η. Dashed lines represent the slope for specific rates of dispersal.

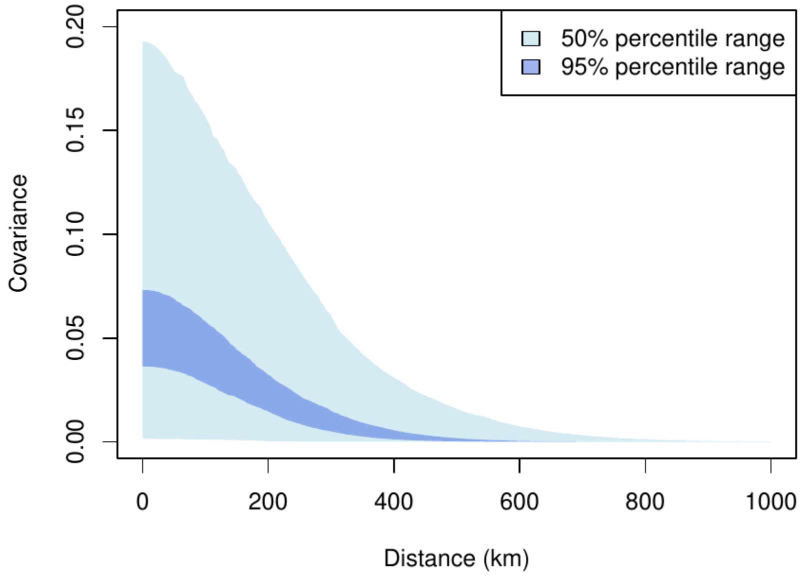

Fig.S8. Prior predictive check for η^2 (modelled as an exponential with a rate of 20) and ρ (modelled as a truncated gamma distribution with a shape of 10 and a rate of 0.06, bounded between 1 and 1350).

2.2 Tactical Simulation

To establish whether the Gaussian Process model described above can recover local variations in dispersal rates, we generated a simulated set of n=150 radiocarbon dates from an equivalent number of sites within our window of analysis. Calendar dates were sampled using a normal distribution (see equations [1], [7-9] above) using the following parameter values: $\varepsilon = 100$; $\beta_0 = 3000$; $\beta_1 = 0.6$; $\rho = 150$; and $\eta^2 = 0.08$. Dates were back-calibrated in ^{14}C age with an error of 20 years.

We fitted our dataset using our Gaussian Process model using the same prior as in our observed dataset and setting τ to 0.9. Model fitting was performed using four chains with 1 million iterations each (half discarded as burn-in, and sampled every 50 steps). MCMC diagnostics showed good convergence, with the highest R-hat (70) equal to 1.003.

Our results indicate that the model does capture the spatial variation in dispersal rates (figure S9), with most of the random effect parameters (figure S10) and all the key fixed effect parameters (figure S11) recovering their respective true values within their 95% quantile intervals.

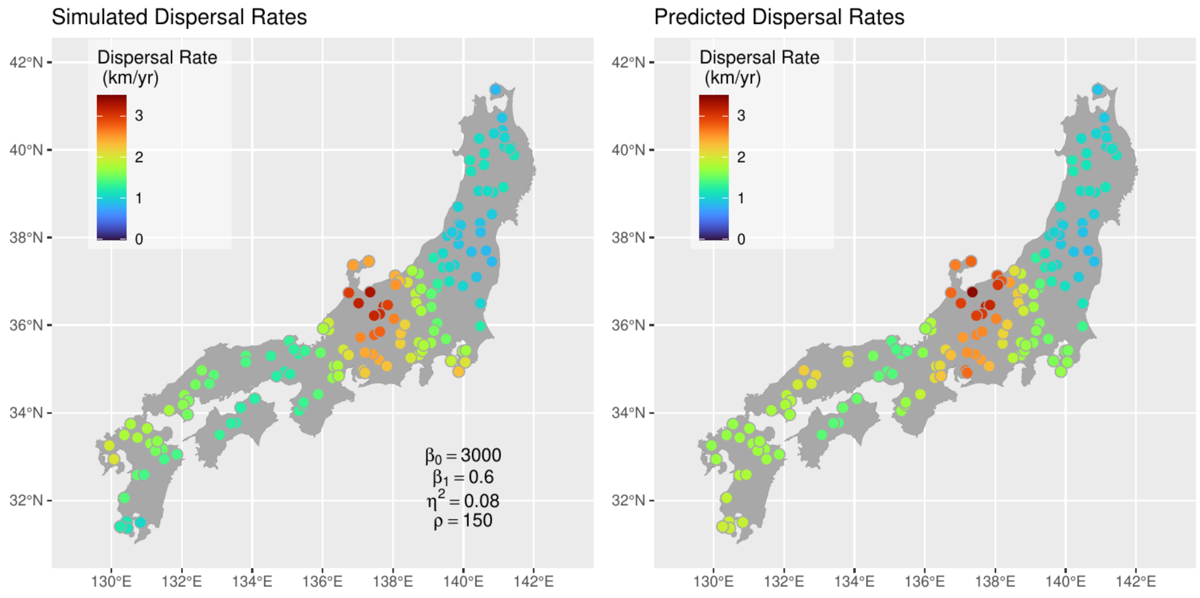

Fig.S9. Simulated and predicted (median posterior) dispersal rates.

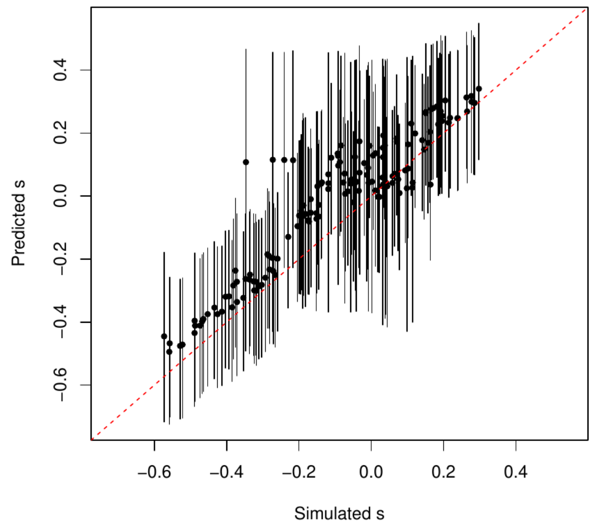

Fig.S10. Simulated and predicted values of the spatial random effect parameter *s*.

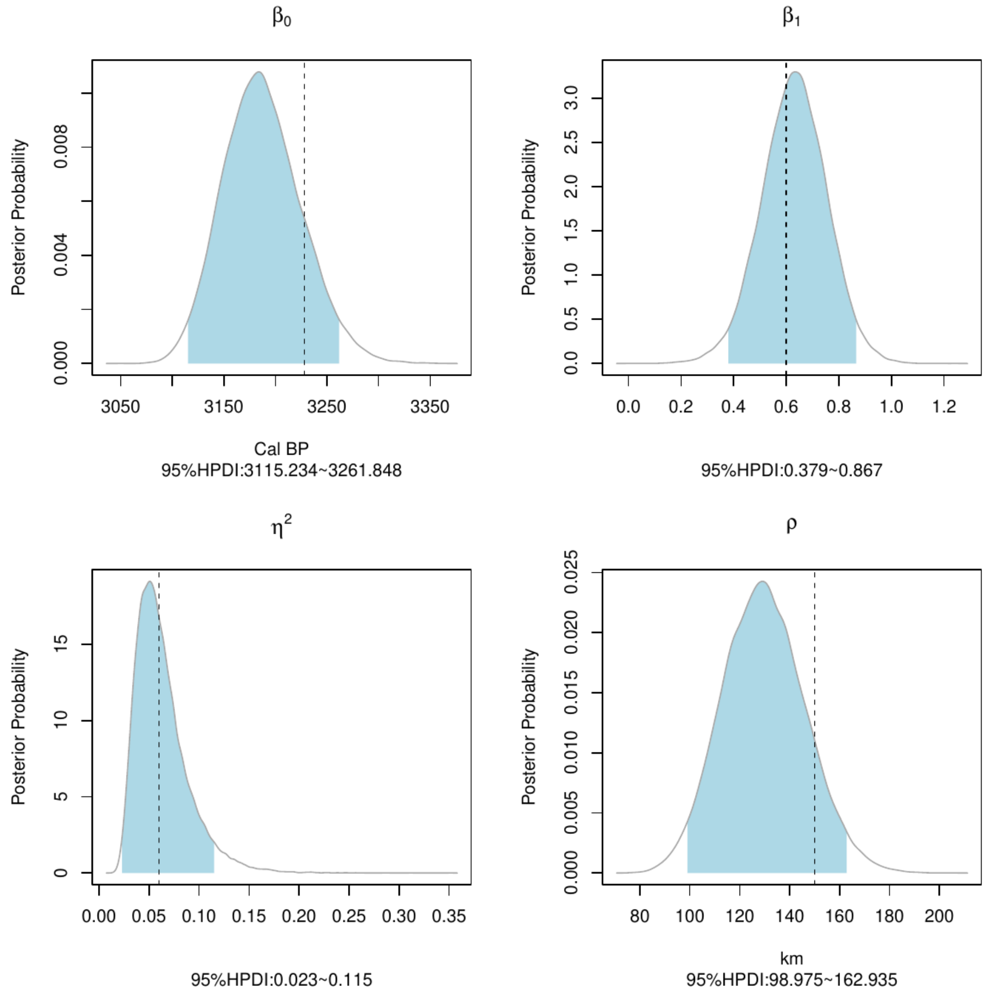

Fig.S11. Posterior distribution of key model parameters for the simulated dataset with corresponding "true" values shown as a dashed line. Notice that in the case of β_0 the line represents the 90th percentile matching the value of τused for inference.

2.3 MCMC Settings, Diagnostics, and Posteriors

As for the non-spatial quantile regression, the model fitting was carried out using the *nimble* and the *nimbleCarbon* R packages. We ran four chains, each with 2 million iterations, half of which were used for burn-in, and values sampled every 100 steps. We used a Metropolis-Hasting adaptive random walk sampler for all parameters except for β_1 and s_i in the GPQR model, which were inferred using an automated factor slice sampler (*71*) to account for correlation between the two parameters. As for the non-spatial quantile regression, the random walker sampler for θ was modified from the default settings (see section 1 above).

The Gelman-Rubin convergence statistics (*70*) were below 1.01 for all parameters, and the trace plots of the four key fixed parameters showed good mixing of the chains (fig. S12 & S13). Figures S14 and S15 show the marginal distribution of these four parameters, whilst tables S1 and S2 provide summary statistics and diagnostics of all fixed parameters.

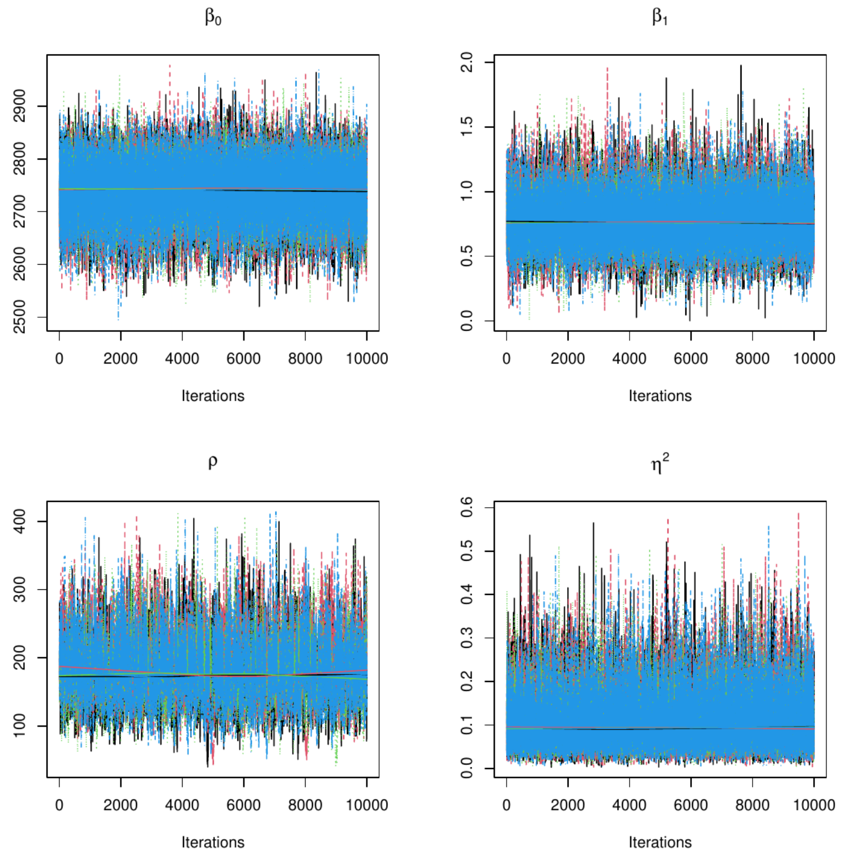

Fig.S12. Traceplots for β_0, β_1, ρ, and η^2 for the τ=0.9 GPQR model.

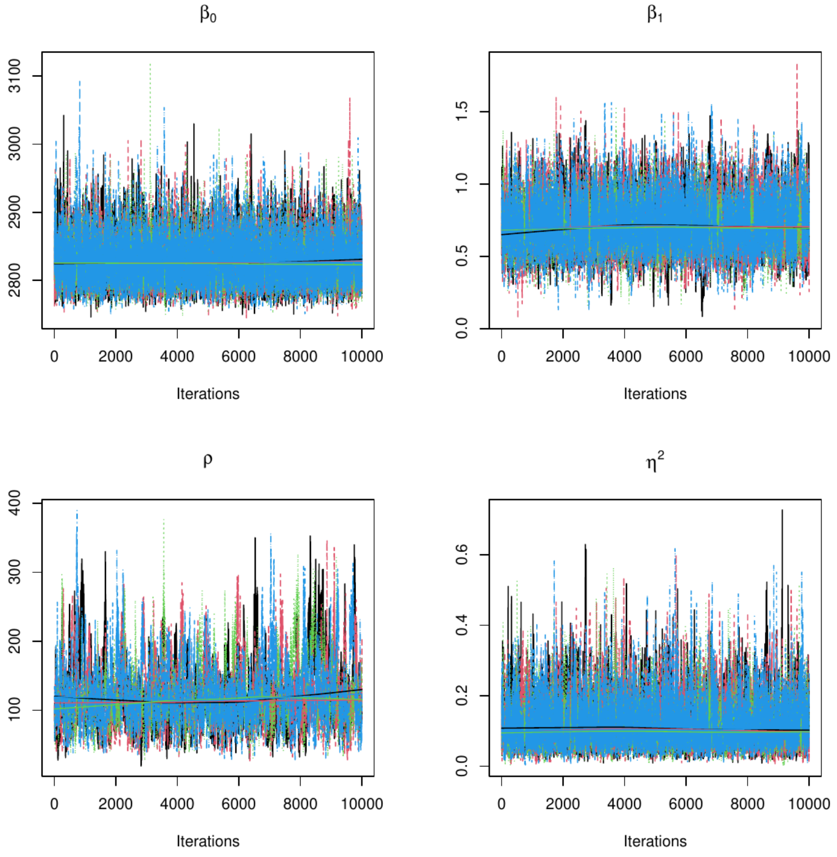

Fig.S13. Trace Plots for β_0, β_1, ρ, and η^2 for the τ=0.99 GPQR model.

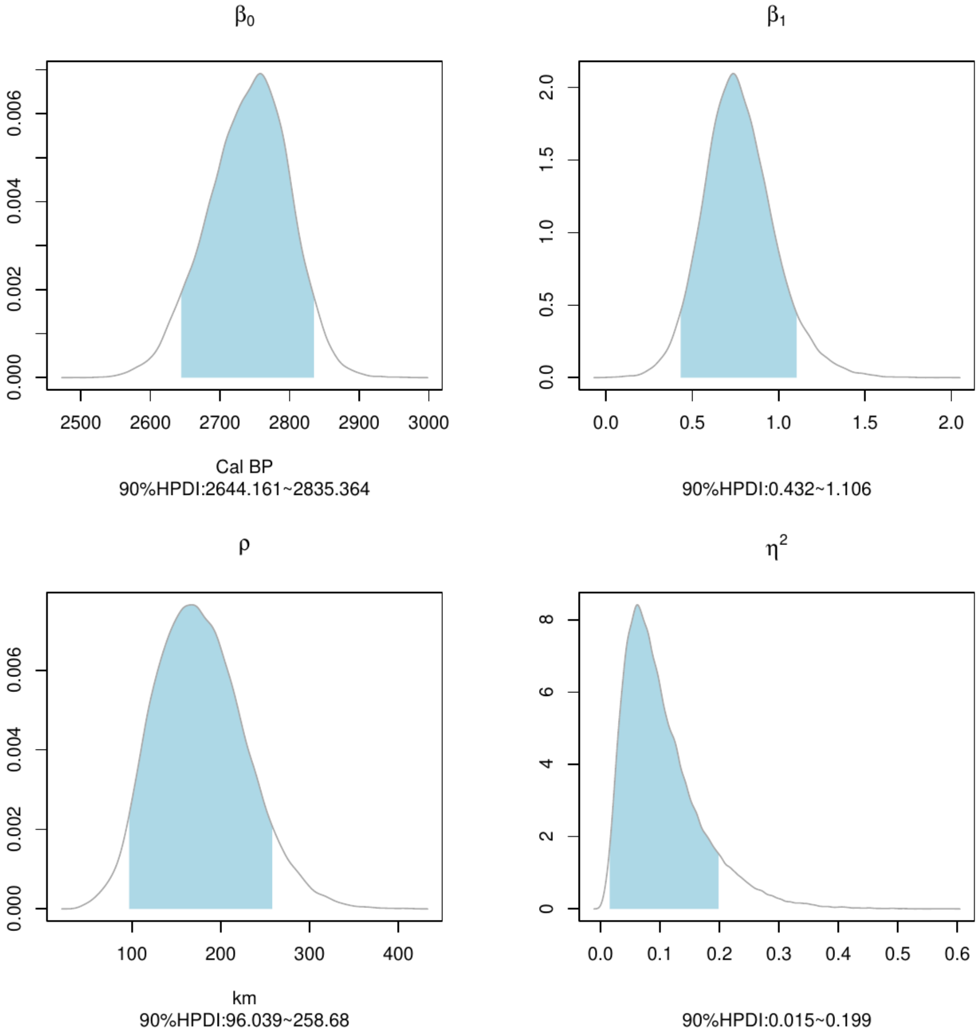

Fig.S14. Marginal posterior distribution of β_0, β_1, ρ, and η^2 in the τ=0.90 GPQR model.

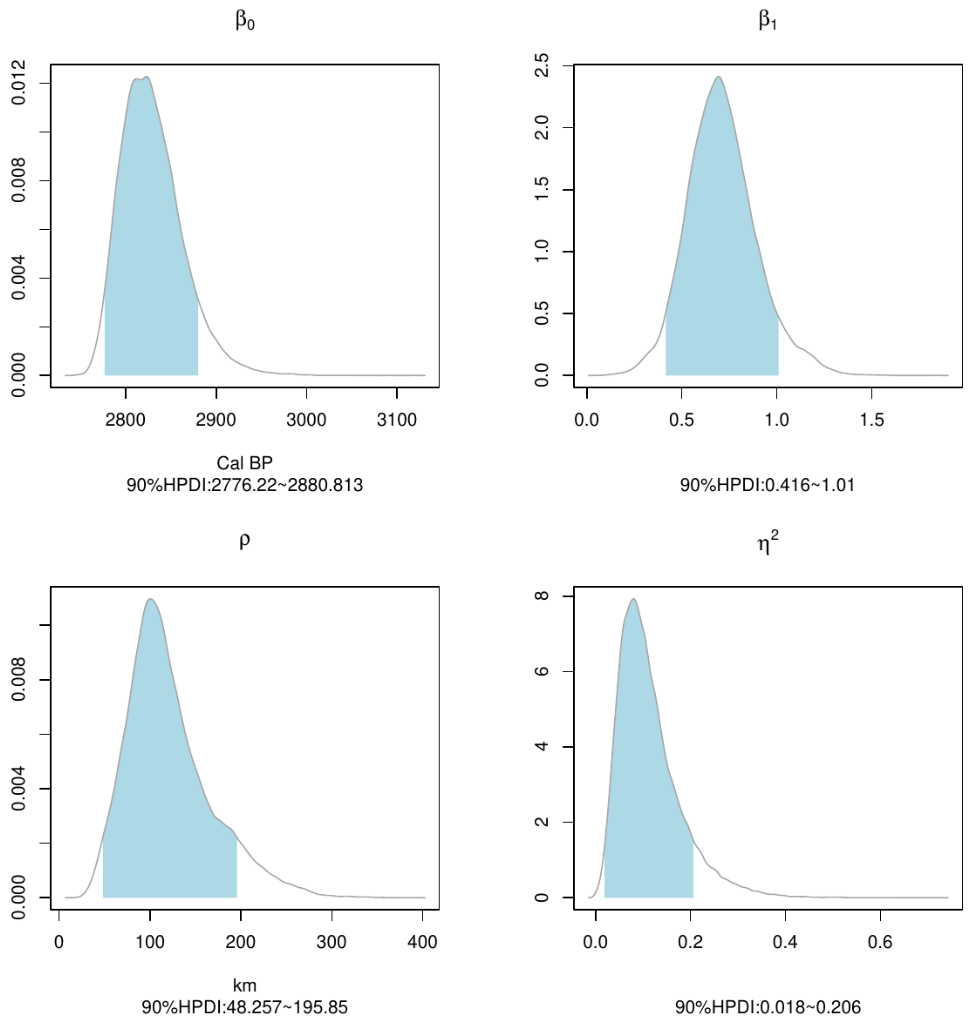

Fig.S15. Marginal posterior distribution of β_0, β_1, ρ, and η^2 in the τ=0.99 GPQR model.

Parameter	Median Posterior	90% HPDI (low)	90% HPDI (high)	R-hat	ESS
β_0	2744	2644	2835	1.00	22266
β_1	0.75	0.43	1.10	1.00	28394
ρ	173.70	96.03	258.68	1.00	3032
η^2	0.0888	0.0147	0.1992	1.00	23224

Table S1 Summary statistics and diagnostics for β_0, β_1, ρ, and η^2 in the τ=0.90 GPQR model.

Parameter	Median Posterior	90% HPDI (low)	90% HPDI (high)	R-hat	ESS
β_0	2825	2776.	2881	1.00	7432
β_1	0.70	0.42	1.01	1.00	2704
ρ	112.32	48.26	195.85	1.01	765
η^2	0.0998	0.0185	0.2063	1.01	3475

Table S2 Summary statistics and diagnostics for β_0, β_1, ρ, and η^2 in the τ=0.99 GPQR model.

3. Bayesian Hierarchical Phase Models with Constraints

Bayesian phase models of radiocarbon dates typically used in stratigraphic contexts have been occasionally applied to study regional phenomena, such as the dating of cultural phases (*72,73*) or the arrival date of populations (*40*) or cultural traits (*74*). These models have been recently used to estimate the arrival dates of crops in different geographic regions (*15,75*), typically employing uniform distribution models as follows:

$$\theta_{i,k} \sim \text{Uniform}(\nu_k, \upsilon_k) \qquad [10]$$

where $\theta_{i,k}$ is the calendar date of sample *i* from Area *k*, and ν_k and υ_k are the start and end dates of the Area *k*. The relationship between $\theta_{i,k}$ and the radiocarbon sample is established using the same procedure described above in the model [2].

The parameter of interest, in this case, is ν_k which provides an estimate of the arrival time of the crop in the focal Area *k*. While there are some interesting non-Bayesian alternatives to this problem (*76*), the advantage of this approach is the possibility to add constraints and priors in multi-regional settings (see below).

A key assumption of this approach, however, is that in order to have unbiased estimates of ν_k and υ_k samples must be randomly and independently selected. For many real statistical applications, this assumption is never truly met, but in the case of crop dispersal, a particular problem arises from the fact that charred remains are often found in distinctive recovery contexts or sites, and as such, samples cannot be regarded as truly independent. A trivial example is to contrast a situation where the inference is carried out on 20 samples from 20 different sites against a situation where the same number of samples are recovered from a single site. In the former case, estimates of ν_k and υ_k are more representative of the start and end dates of a region, but in the latter case, these would represent the start and end date of the particular site from which the samples are taken. While such extreme situations are rare, multiple samples are frequently taken from the same site or context and as such, might represent a substantial portion of the dates from a particular region. For example, Leipe and colleagues (*15*) estimated the arrival date of rice farming in the Japanese central highlands using 32 dates, but ca. 40% of them (n=13) were from a single site (Chikarishijori).

One way to approach this problem and to take into account the fact that samples are typically recovered from the same site is to employ a hierarchical model:

$$\theta_{i,j,k} \sim \text{Uniform}(\alpha_{j,k}, \alpha_{j,k} + \delta_{j,k}) \qquad [11]$$
$$\alpha_{j,k} \sim \text{Uniform}(\nu_k, \upsilon_k) \qquad [12]$$

where $\alpha_{j,k}$ is the start date of crop use at site *j* in region *k*, and $\delta_{j,k}$ is the duration of rice farming in the same site. Inference of the regional start date is then no longer based on the individual charred dates $\theta_{i,j,k}$ but instead from the start date of individual sites. This effectively acknowledges sample independence; sites with a larger number of dates will still provide more information, but mainly in terms of having more accurate estimates of $\alpha_{j,k}$. From an interpretative point, [12] is slightly different from [10] as the former represents the distribution of start dates at each site while the latter represents just the distribution of dates within the area *k*. However, the interpretation of our primary parameter of interest (i.e. ν_k) as the arrival time of the crop in the practical area remains the same. Model [11] is

parameterised in a way so that the end date is effectively defined by the duration parameter $\delta_{j,k}$. Inference of this parameter is actually helped by the presence of sites yielding multiple dates and by the choice of some weakly informative prior that conveys assumptions on site duration. Of course, if the entirety of the dataset consists of one date per site, then there is effectively no way to infer $\delta_{j,k}$, but under those circumstances, arrival dates can be safely estimated using model [10].

3.1 Tactical Simulation

To illustrate the difference between the standard model [10] and the proposed hierarchical modification depicted in [11] and [12], we simulated an artificial archaeological dataset consisting of 30 radiocarbon dates distributed exponentially across 10 archaeological sites within a single region ($n_1 = 13$, $n_2 = 3$, $n_3 = 5$, $n_4 = 3$, $n_5 = n_6 = n_7 = n_8 = n_9 = n_{10} = 1$). Start dates of occupation (i.e. α_j) were modelled using a uniform distribution with regional start and end dates ν and υ dates equal to 3500 and 3000 cal BP. The site duration parameter δ_j was randomly sampled from a gamma distribution with shape parameter 5 and rate parameter 0.02. The simulated calendar dates were back-calibrated in ^{14}C age and assigned to an error of 20 years.

We estimated ν and υ using both the conventional non-hierarchical approach (model [10]) and the proposed solution (model [11-12]) using three chains and 2 million iterations (half discarded for burnin and sampled every 100 steps). For model [10] we used a uniform prior for ν and υ between 0 and 10000, with the constraint ν > υ (i.e. ν earlier than υ). For model [11-12] we additionally modelled δ_j using a uniform hyper-prior for the rate (bounded between 1 and 20) and a normal distribution with a mean 200 and standard deviation 100 truncated between 1 and 500 for the mode, where the rate is equal to (shape-1)/mode. MCMC diagnostic showed good convergence for all parameters (Rhat < 1.01).

Figure S16 shows the difference in the marginal posterior distribution of ν and υ in the two models. The conventional model has a narrower posterior given the inference is made on 30 samples, whilst the hierarchical model has a wider posterior as the inference is primarily driven on the sites. However, the posterior of the conventional model is less accurate and fails to capture the true start date (dashed line), which is instead recovered by the hierarchical model.

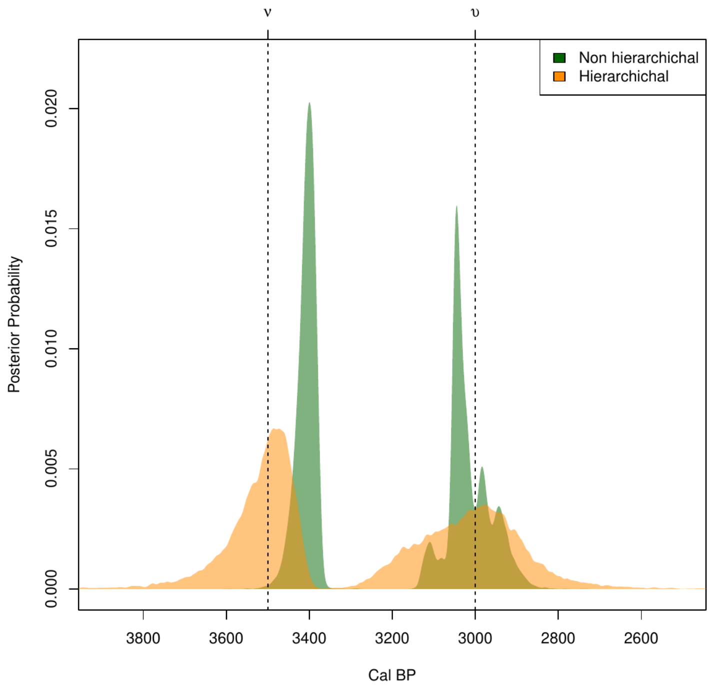

Fig.S16. Marginal posterior distribution of the stard and end dates ν and υ in the hierarchical and non-hierarchical models .

3.2 Implementation details for the observed data

The hierarchical phase model described in section 3.1 was fitted to the charred rice dates from the eight geographic areas (table S3), whose boundaries were informed by previous archaeological studies and the results of our GPQR analysis, to ensure that dispersal rates within each area were as consistent as possible. We particularly made an effort to avoid having instances where a major change in dispersal rate occurred within each area so that an early estimate of arrival date was the consequence of dates occurring at sites located at the boundary of adjacent areas.

Area	Prefectures	Number of Dates	Number of Sites
I	Saga | Fukuoka | Nagasaki	32	19
II	Miyazaki | Oita | Kumamoto |Kagoshima	49	22
III	Ehime | Kochi | Hiroshima | Okayama | Tottori | Shimane | Yamaguchi	22	14
IV	Hyogo | Kagawa | Kyoto | Mie | Nara | Osaka | Shiga | Tokushima | Wakayama	32	11
V	Aichi | Gifu | Ishikawa | Fukui | Kanagawa | Niigata | Nagano | Toyama | Shizuoka | Yamanashi	94	35
VI	Chiba | Gunma | Ibaraki | Tokyo | Saitama | Tochigi	38	14
VII	Akita | Fukushima | Iwate | Miyagi | Yamagata	18	9

VIII	Aomori	9	8
TOTAL		294	132

Table S3. Number of sites and radiocarbon dates from each area

Priors of the start and end dates ν and υ were based on a uniform distribution bounded between 500 and 5000 cal BP, with the constraint $\nu > \upsilon$. The duration parameter δ was modelled using a gamma distribution, with the shape and rate parameters modelled using a uniform hyper-prior for the rate (bounded between 1 and 20) and a normal distribution with a mean 200 and standard deviation 100 truncated between 1 and 500 for the mode, where the rate is equal to (shape-1)/mode. Figure S17 shows the prior predictive check with these settings and illustrates how our model includes a wide range of possible settlement durations.

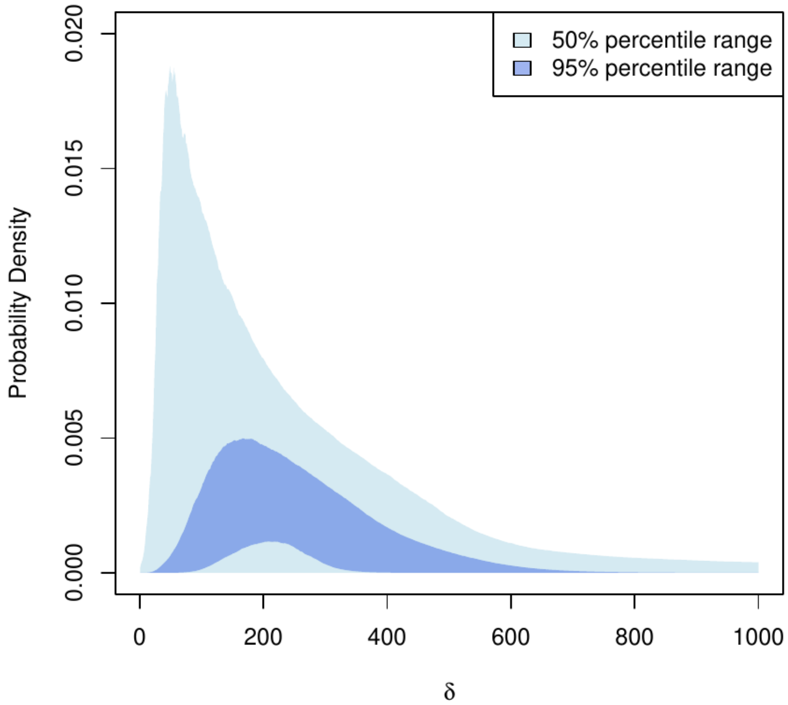

Fig.S17. Prior predictive check for the duration parameter δ. Generating using 5,000 random samples from relevant priors.

We considered two different versions of the hierarchical model. In **model a**, we infer all parameters without any constraints, hence allowing for all possible temporal orders of our primary parameter of interest ν. This allows us to infer the arrival date of rice in each area on the exclusive basis of the dates from that focal area. In **model b**, we add the following constraints: $\nu_{I} > \nu_{kII}$, $\nu_{I} > \nu_{III} > \nu_{IV} > \nu_{V}$, $\nu_{IV} > \nu_{VI}$, $\nu_{IV} > \nu_{VII}$, and $\nu_{IV} > \nu_{VIII}$. This effectively incorporates the assumption of a wave-of-advance-like model originating from northern Kyushu (Area I), whilst allowing for possible leap-frogging after the dispersal reached the Kansai area (Area IV).

Both models were fitted using 6 million iterations, half of which were dedicated to burn-in, and sampled every 300 steps. We used Metropolis-Hasting adaptive random walk sampler for all parameters using default settings, with the exception of the age estimates θ, where we used an

adaptInterval of 20,000 and an *adaptFactorExponent* to 0.1. Model convergence was assessed via visual inspections of the trace plots and Gelman-Rubin convergence statistics. All parameters yielded an R-hat below 1.01, with the exception of one θ_{166} which returned an Rhat of 1.03 in **model b.** Visual inspection of the trace plot indicated that this was most likely due to the multi-modal shape of the posterior. The high agreement index (98.4, (77)), as well as comparison with the associated calibrated date, suggests that there are no concerns from an inferential point of view.

3.3 Posterior Distributions

Table S4 shows the summary statistics of the arrival date estimates ν for the two models, whilst figures S18 and S19 show their marginal posterior distribution. Figure S20 indicates the probability that ν values of row areas are smaller (i.e. later) than column areas, with cells coloured in blue supporting a wave-of-advance scenario, while those coloured in red indicate possible leap-frog dispersals. Finally, figure S21 shows the pairwise difference in ν (column area minus row area) obtained from the posterior samples, with portions of the 90% highest posterior density interval coloured in blue for positive values (column earlier than row) and in red for negative values (row earlier than column).

Model	Area	Median Posterior	90% HPDI (low)	90% HPDI (high)	Rhat	ESS
Model a	I	986 BC	1176 BC	845 BC	1	39981
	II	572 BC	740 BC	430 BC	1	39424
	III	887 BC	1176 BC	669 BC	1.001	11796
	IV	905 BC	1179 BC	695 BC	1	30453
	V	655 BC	771 BC	552 BC	1	38439
	VI	273 BC	478 BC	122 BC	1	39026
	VII	153 BC	458 BC	44 AD	1	40000
	VIII	469 BC	943 BC	177 BC	1	38182
Model b	I	1039 BC	1251 BC	872 BC	1	39658
	II	570 BC	735 BC	430 BC	1	40326
	III	910 BC	1061 BC	779 BC	1	30742
	IV	824 BC	946 BC	703 BC	1	23067
	V	648 BC	754 BC	560 BC	1	36917
	VI	271 BC	471 BC	124 BC	1	35089
	VII	152 BC	434 BC	42 AD	1	39185
	VIII	428 BC	709 BC	203 BC	1	37394

Table S4. Summary statistics and diagnostics for the ν parameters in the two models.

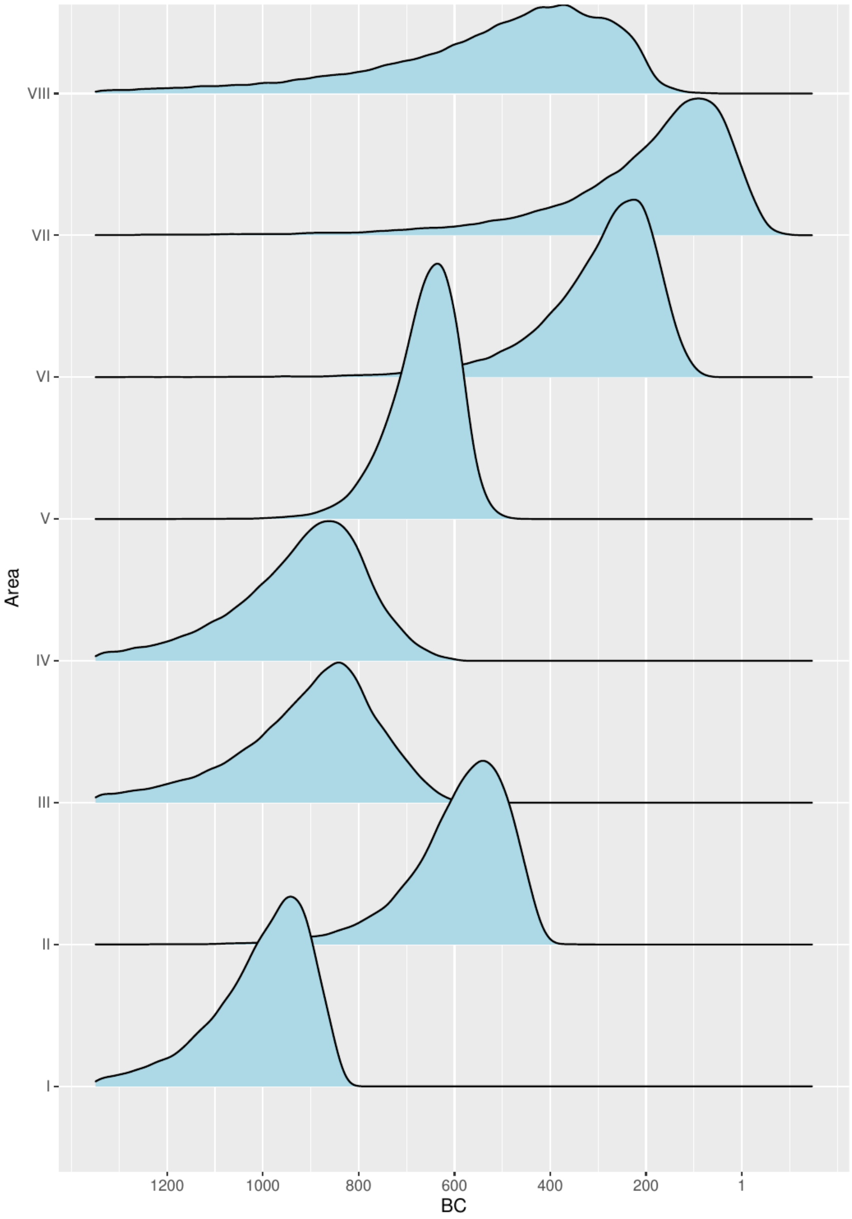

Fig.S18. Marginal posterior distribution of ν for model a

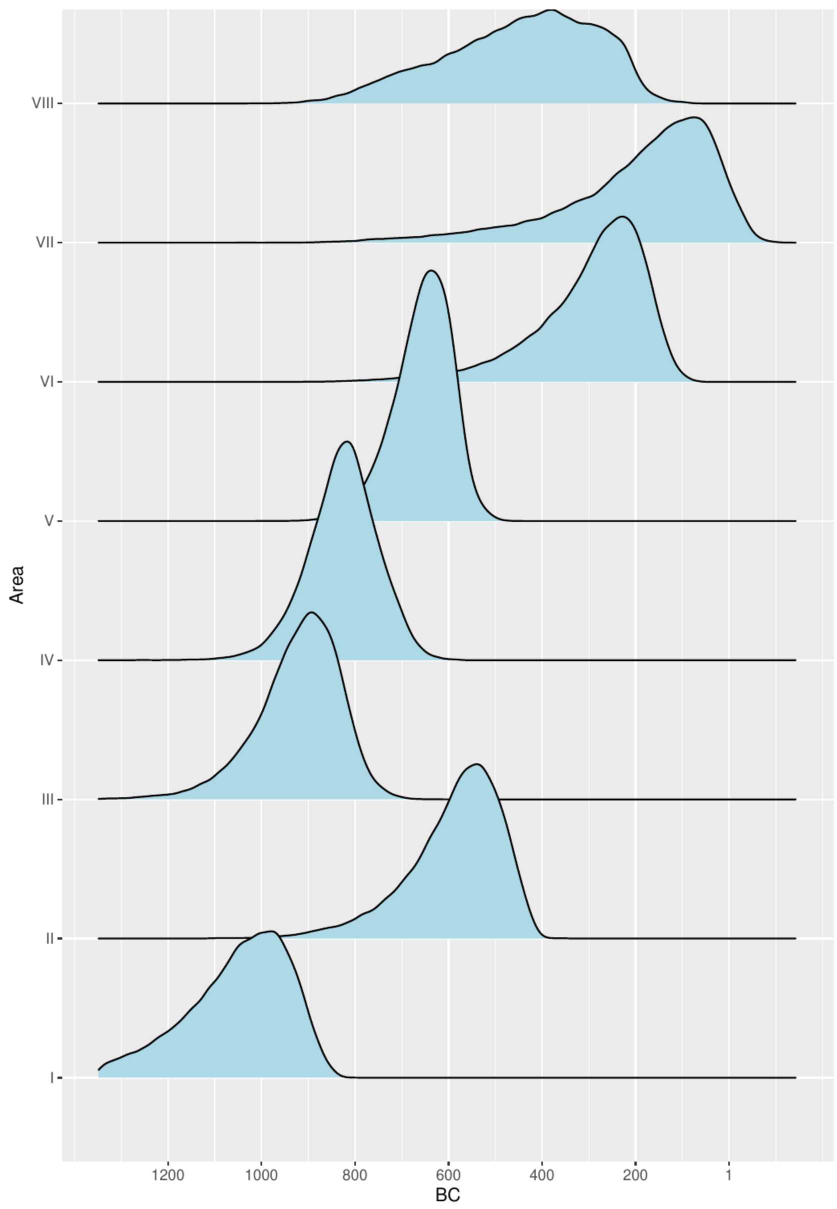

Fig.S19. Marginal posterior distribution of ν for model b

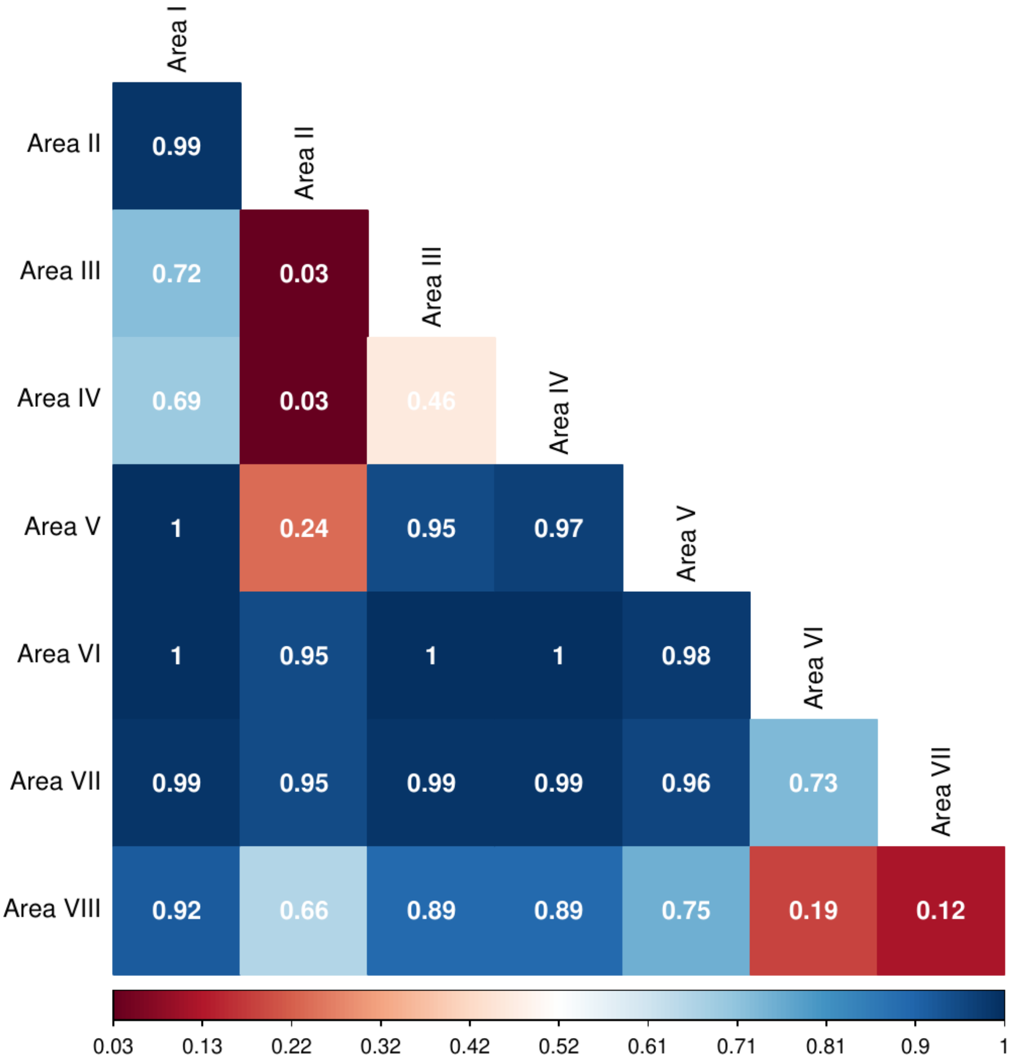

Fig.S20. Probability of row areas's v being after colum area's v for model a

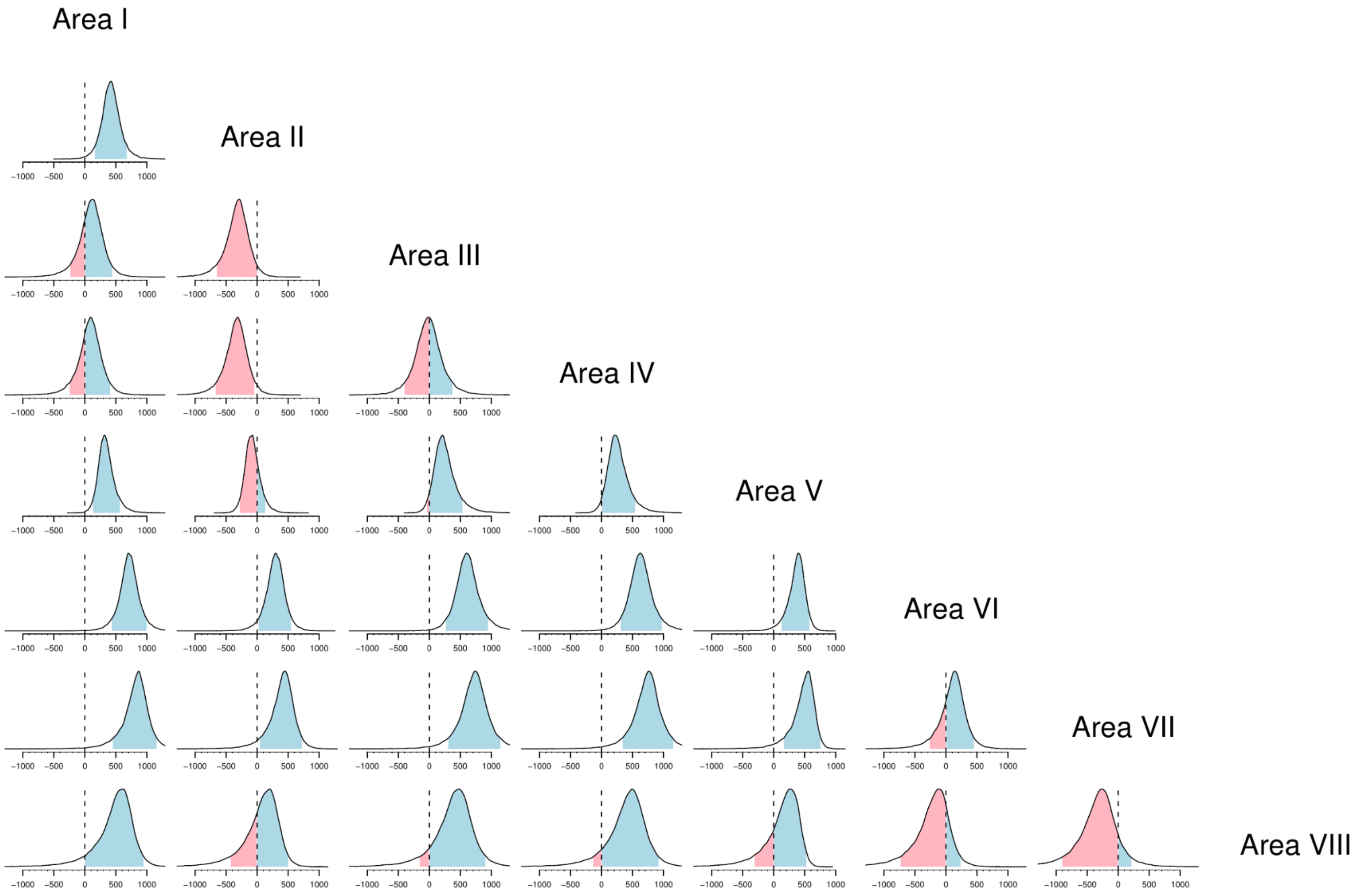

Fig.S21. Posterior estimates of pairwise difference (column minus row) of ν in model a. Highlighted regions represent the 90% HPDI (red: row is earlier; blue: row is later)

REFERENCES AND NOTES

1. J. Diamond, P. Bellwood, Farmers and their languages: The first expansions. *Science* **300**, 597–603 (2003).

2. P. Bellwood, *First Farmers: The Origins of Agricultural Societies* (John Wiley & Sons, 2004).

3. L. L. Cavalli-Sforza, P. Menozzi, A. Piazza, Demic expansions and human evolution. *Science* **259**, 639–646 (1993).

4. J.-P. Bocquet-Appel, S. Naji, M. V. Linden, J. K. Kozlowski, Detection of diffusion and contact zones of early farming in Europe from the space-time distribution of 14C dates. *J. Archaeol. Sci.* **36**, 807–820 (2009).

5. J. Fort, Synthesis between demic and cultural diffusion in the Neolithic transition in Europe. *Proc. Natl. Acad. Sci. U.S.A.* **109**, 18669–18673 (2012).

6. J. G. de Souza, J. A. Mateos, M. Madella, Archaeological expansions in tropical South America during the late Holocene: Assessing the role of demic diffusion. *PLOS ONE* **15**, e0232367 (2020).

7. A. J. Ammerman, L. L. Cavalli-Sforza, Measuring the rate of spread of early farming in Europe. *Man* **6**, 674–688 (1971).

8. M. Gkiasta, T. Russell, S. Shennan, J. Steele, Neolithic transition in Europe: The radiocarbon record revisited. *Antiquity* **77**, 45–62 (2003).

9. R. Pinhasi, J. Fort, A. J. Ammerman, Tracing the origin and spread of agriculture in Europe. *PLOS Biol.* **3**, e410 (2005).

10. F. Silva, J. Steele, New methods for reconstructing geographical effects on dispersal rates and routes from large-scale radiocarbon databases. *J. Archaeol. Sci.* **52**, 609–620 (2014).

11. F. Silva, A. Weisskopf, C. Castillo, C. Murphy, E. Kingwell-Banham, L. Qin, D. Q. Fuller, A tale of two rice varieties: Modelling the prehistoric dispersals of *japonica* and proto-*indica* rices. *Holocene* **28**, 1745–1758 (2018).

12. L. Betti, R. M. Beyer, E. R. Jones, A. Eriksson, F. Tassi, V. Siska, M. Leonardi, P. Maisano Delser, L. K. Bentley, P. R. Nigst, J. T. Stock, R. Pinhasi, A. Manica, Climate shaped how Neolithic farmers and European hunter-gatherers interacted after a major slowdown from 6,100 bce to 4,500 bce. *Nat. Hum. Behav.* **4**, 1004–1010 (2020).

13. N. Isern, J. Fort, M. Vander Linden, Space competition and time delays in human range expansions. Application to the neolithic transition. *PLOS ONE* **7**, e51106 (2012).

14. J. Fort, Demic and cultural diffusion propagated the Neolithic transition across different regions of Europe. *J. R. Soc. Interface* **12**, 20150166 (2015).

15. C. Leipe, T. Long, M. Wagner, T. Goslar, P. E. Tarasov, The spread of rice to Japan: Insights from Bayesian analysis of direct radiocarbon dates and population dynamics in East Asia. *Quat. Sci. Rev.* **244**, 106507 (2020).

16. M. Porčić, T. Blagojević, J. Pendić, S. Stefanović, The timing and tempo of the Neolithic expansion across the Central Balkans in the light of the new radiocarbon evidence. *J. Archaeol. Sci. Rep.* **33**, 102528 (2020).

17. H. Kanaseki, M. Sahara, The Yayoi Period. *Asian Perspect.* **19**, 15–26 (1976).

18. K. Mizoguchi, *The Archaeology of Japan: From the Earliest Rice Farming Villages to the Rise of the State* (Cambdrige Univ. Press, 2013).

19. G. W. Crawford, Advances in understanding early agriculture in Japan. *Curr. Anthropol.* **52**, S331–S345 (2011).

20. H. Nasu, Domestication of plants during the Jomon period. *Daiyonki kenkyū (The Quaternary Research).* **57**, 109–126 (2018).

21. M. J. Hudson, S. Nakagome, J. B. Whitman, The evolving Japanese: The dual structure hypothesis at 30. *Evol. Hum. Sci.* **2**, E6 (2020).

22. S. Nakagome, T. Sato, H. Ishida, T. Hanihara, T. Yamaguchi, R. Kimura, S. Mano, H. Oota; Asian DNA Repository Consortium, Model-based verification of hypotheses on the origin of modern Japanese revisited by Bayesian inference based on genome-wide SNP data. *Mol. Biol. Evol.* **32**, 1533–1543 (2015).

23. K. Miyamoto, The spread of rice agriculture during the Yayoi Period: From the Shandong Peninsula to the Japanese Archipelago via the Korean Peninsula. *Jpn. J. Archaeol.* **6**, 109–124 (2019).

24. K. Kobayashi, in *Yayoinōko no hajimari to sono nendai*, T. Nishimoto, Ed. (Yuzankaku, 2009), *Yayoijidai no hajimari to sono nendai*, pp. 55–82.

25. S. Fujio, The frame of the Yayoi Culture: Is wet rice cultivation with irrigation system an indicator of the Yayoi culture? *Bull. Natl. Museum Jpn. Hist.* **178**, 85–120 (2013).

26. S. Kobayashi, in *Yayoijidai wa dō kawaruka: tanso14nendai to atarashii kodaizō wo motomete*, K. Hirose, Ed. (Gakuseisha, 2007), *Rekihaku forum*, pp. 136–157.

27. S. Fujio, Early grain cultivation and starting processes in the Japanese archipelago. *Quaternary* **4**, 3 (2021).

28. K. Miyamoto, A new discussion of the actual date of the beginning of the Yayoi period. *Kokogaku Zasshi.* **100**, 1–27 (2018).

29. S. Nakayama, *Shokubutsukōkogaku to Nihon no nokō no kigen* (Dōseisha, 2010).

30. A. Jarosz, M. Robbeets, R. Fernandes, H. Takamiya, A. Shinzato, N. Nakamura, M. Shinoto, M. Hudson, Demography, trade and state power: A tripartite model of medieval farming/language dispersals in the Ryukyu Islands. *Evol. Hum. Sci.* **4**, E4 (2022).

31. K. Fujino, Y. Hirayama, R. Kaji, Marker-assisted selection in rice breeding programs in Hokkaido. *Breed. Sci.* **69**, 383–392 (2019).

32. S. Koyama, Jomon subsistence and population. *Senri Ethnol. Stud.* **2**, 1–65 (1978).

33. S. Kobayashi, Eastern Japanese pottery during the Jomon-Yayoi transition: A study in forager-farmer interaction. *Bull. Indo-Pacific Preshist. Assoc.* **21**, 37–42 (2001).

34. S. Fujio, When did the wet rice cultivation with the irrigation system begin in the Western Japan. *Bull. Natl. Museum Jpn. Hist.* **183**, 113–143 (2014).

35. E. Endo, C. Leipe, The onset, dispersal and crop preferences of early agriculture in the Japanese archipelago as derived from seed impressions in pottery. *Quat. Int.* **623**, 35–49 (2022).

36. T. Long, M. Wagner, P. E. Tarasov, A Bayesian analysis of radiocarbon dates from prehistoric sites in the Haidai Region, East China, for evaluation of the archaeological chronology. *J. Archaeol. Sci. Rep.* **12**, 81–90 (2017).

37. D. Filipović, J. Meadows, M. D. Corso, W. Kirleis, A. Alsleben, Ö. Akeret, F. Bittmann, G. Bosi, B. Ciută, D. Dreslerová, H. Effenberger, F. Gyulai, A. G. Heiss, M. Hellmund, S. Jahns, T. Jakobitsch, M. Kapcia, S. Klooß, M. Kohler-Schneider, H. Kroll, P. Makarowicz, E. Marinova, T. Märkle, A. Medović, A. M. Mercuri, A. Mueller-Bieniek, R. Nisbet, G. Pashkevich, R. Perego, P. Pokorný, Ł. Pospieszny, M. Przybyła, K. Reed, J. Rennwanz, H.-P. Stika, A. Stobbe, T. Tolar, K. Wasylikowa, J. Wiethold, T. Zerl, New AMS ^{14}C dates track the arrival and spread of broomcorn millet cultivation and agricultural change in prehistoric Europe. *Sci. Rep.* **10**, 13698 (2020).

38. J. van Etten, R. J. Hijmans, A geospatial modelling approach integrating archaeobotany and genetics to trace the origin and dispersal of domesticated plants. *PLOS ONE* **5**, e12060 (2010).

39. F. Silva, C. J. Stevens, A. Weisskopf, C. Castillo, L. Qin, A. Bevan, D. Q. Fuller, Modelling the geographical origin of rice cultivation in asia using the rice archaeological database. *PLOS ONE* **10**, e0137024 (2015).

40. P. G. Blackwell, C. E. Buck, The Late Glacial human reoccupation of north-western Europe: New approaches to space-time modelling. *Antiquity* **77**, 232–240 (2003).

41. E. E. Cochrane, T. M. Rieth, D. Filimoehala, The first quantitative assessment of radiocarbon chronologies for initial pottery in Island Southeast Asia supports multi-directional Neolithic dispersal. *PLOS ONE* **16**, e0251407 (2021).

42. A. Jerardino, J. Fort, N. Isern, B. Rondelli, Cultural diffusion was the main driving mechanism of the neolithic transition in Southern Africa. *PLOS ONE* **9**, e113672 (2014).

43. P. Riris, F. Silva, Resolution and the detection of cultural dispersals: Development and application of spatiotemporal methods in Lowland South America. *Humanit. Soc. Sci. Commun.* **8**, 36 (2021).

44. M. J. Hamilton, B. Buchanan, Spatial gradients in Clovis-age radiocarbon dates across North America suggest rapid colonization from the north. *Proc. Natl. Acad. Sci. U.S.A.* **104**, 15625–15630 (2007).

45. M. Hudson, *Ruins of Identity: Ethnogenesis in the Japanese Islands* (University of Hawai'i Press, 1999).

46. E. de Boer, M. A. Yang, A. Kawagoe, G. L. Barnes, Japan considered from the hypothesis of farmer/language spread. *Evol. Hum. Sci.* **2**, E13 (2020).

47. M. Nakazawa, T. Ushino, Repurikahō ni yoru San'inchihō Jōmonjidai-banki doki no momijō-akkonne no kansatsu. *Jōmon jidai.* **14**, 139–153 (2003).

48. T. Hamada, in *Nōkōbunkafukugōkeisei no kōkogaku: Nōkō no hajimari*, H. Shitara, Ed. (Yuzankaku, 2019), vol. 1, pp. 141–160.

49. T. Kamimura, *Hayato no Kōkogaku* (Nyū saiensu sha, 1984).

50. F. Oda, in *Nihon no Kōkogaku IV Kofunjidai (ue)*, Y. Endo, C. Fujisawa, Eds. (Kawade shobō, 1966), pp. 114–174.

51. H. Takamiya, N. Nakamura, The beginning of agriculture in the Ryukyu Archipelago. *South Pacific Studies* **41**, 1–34 (2021).

52. Y. Sasaki, S. Noshiro, in *Nokobunkafuugokeisei no koukogaku (ue)*, H. Shitara, Ed. (Yuzankaku, 2019), pp. 127–142.

53. G. L. Barnes, The Jōmon–Yayoi transition in eastern Japan: Enquiries from the Kantō Region. *Jpn. J. Archaeol.* **7**, 1–51 (2019).

54. H. Shitara, in *Inasakudenrai*, S. Nakazono, H. Morioka, H. Shitara, Eds. (Iwanami Shoten, 2004).

55. K. Takase, in *Yayoi-jidaitte dona jidai dattanoka?*, S. Fujio, Ed. (Asakurashoten, 2017), pp. 114–136.

56. K. Hayashi, in *Nihonkōkogaku: bunka to chiikisei*, M. Tozawa, Y. Kondo, M. Anbiru, K. Hayashi, N. Shimojo, Y. Fukasawa, N. Takei, K. Amakasu, T. Kubo, M. Yoshizaki, S. Kin, S. Touma, Eds. (Iwanami Shoten, 1985), *Iwanamikōza*, pp. 93–124.

57. T. Akazawa, in *Hunters in transition: Mesolithic societies of temperate Eurasia ad their transition to farming*, M. Zvelebil, Ed. (Cambridge Univ. Press, 1986), pp. 151–165.

58. Y. Kudo, M. Sakamoto, M. Hakozaki, Approach for creating database of the radiocarbon dates published on the archaeological research reports in Japan. *Bull. Natl. Museum Jpn. Hist.* **212**, 251–266 (2018).

59. P. J. Reimer, W. E. N. Austin, E. Bard, A. Bayliss, P. G. Blackwell, C. B. Ramsey, M. Butzin, H. Cheng, R. L. Edwards, M. Friedrich, P. M. Grootes, T. P. Guilderson, I. Hajdas, T. J. Heaton, A. G. Hogg, K. A. Hughen, B. Kromer, S. W. Manning, R. Muscheler, J. G. Palmer, C. Pearson, J. van der Plicht, R. W. Reimer, D. A. Richards, E. M. Scott, J. R. Southon, C. S. M. Turney, L. Wacker, F. Adolphi, U. Büntgen, M. Capano, S. M. Fahrni, A. Fogtmann-Schulz, R. Friedrich, P. Köhler, S. Kudsk, F. Miyake, J. Olsen, F. Reinig, M. Sakamoto, A. Sookdeo, S. Talamo, The IntCal20 Northern hemisphere radiocarbon age calibration curve (0–55 cal kBP). *Radiocarbon* **62**, 725–757 (2020).

60. K. Yu, R. A. Moyeed, Bayesian quantile regression. *Stat. Probab. Lett.* **54**, 437–447 (2001).

61. P. de Valpine, D. Turek, C. J. Paciorek, C. Anderson-Bergman, D. T. Lang, R. Bodik, Programming with models: Writing statistical algorithms for general model structures with NIMBLE. *J. Comput. Graph. Stat.* **26**, 403–413 (2017).

62. P. de Valpine, C. Adler, D. Turek, N. Michaud, C. Anderson-Bergman, F. Obermeyer, C. Wehrhahn Cortes, A. Rodrìguez, D. Temple Lang, S. Paganin, *NIMBLE: MCMC, Particle Filtering, and Programmable Hierarchical Modeling* (2020); https://doi.org/10.5281/zenodo.1211190.

63. E. R. Crema, nimbleCarbon: Models and Utility Functions for Bayesian Analyses of Radiocarbon Dates with NIMBLE (v.0.2.0) (2022); https://github.com/ercrema/nimbleCarbon.

64. E. R. Crema, S. Shoda, A Bayesian approach for fitting and comparing demographic growth models of radiocarbon dates: A case study on the Jomon-Yayoi transition in Kyushu (Japan). *PLOS ONE* **16**, e0251695 (2021).

65. J. Steele, Radiocarbon dates as data: Quantitative strategies for estimating colonization front speeds and event densities. *J. Archaeol. Sci.* **37**, 2017–2030 (2010).

66. C. E. Buck, C. D. Litton, A. F. M. Smith, Calibration of radiocarbon results pertaining to related archaeological events. *J. Archaeol. Sci.* **19**, 497–512 (1992).

67. R Core Team, *R: A Language and Environment for Statistical Computing* (R Foundation for Statistical Computing, Vienna, Austria, 2020); www.R-project.org/.

68. R. Koenker, quantreg: Quantile Regression. R package version 5.86 (2021); https://CRAN.R-project.org/package=quantreg.

69. C. E. Rasmussen, C. K. I. Williams, *Gaussian Processes for Machine Learning* (MIT Press, 2005).

70. A. Gelman, D. B. Rubin, Inference from iterative simulation using multiple sequences. *Stat. Sci.* **7**, 457–472 (1992).

71. M. M. Tibbits, C. Groendyke, M. Haran, J. C. Liechty, Automated factor slice sampling. *J. Comput. Graph. Stat.* **23**, 543–563 (2014).

72. M. Brunner, J. von Felten, M. Hinz, A. Hafner, Central European Early Bronze Age chronology revisited: A Bayesian examination of large-scale radiocarbon dating. *PLOS ONE* **15**, e0243719 (2020).

73. E. R. Crema, K. Kobayashi, A multi-proxy inference of Jōmon population dynamics using bayesian phase models, residential data, and summed probability distribution of ^{14}C dates. *J. Archaeol. Sci.* **117**, 105136 (2020).

74. G. Capuzzo, J. A. Barceló, Cultural changes in the second millennium BC: A Bayesian examination of radiocarbon evidence from Switzerland and Catalonia. *World Archaeol.* **47**, 622–641 (2015).

75. T. Long, C. Leipe, G. Jin, M. Wagner, R. Guo, O. Schröder, P. E. Tarasov, The early history of wheat in China from 14C dating and Bayesian chronological modelling. *Nat. Plants.* **4**, 272–279 (2018).

76. A. J. M. Key, D. L. Roberts, I. Jarić, Statistical inference of earlier origins for the first flaked stone technologies. *J. Hum. Evol.* **154**, 102976 (2021).

77. C. Bronk Ramsey, Radiocarbon calibration and analysis of stratigraphy: The OxCal program. *Radiocarbon* **37**, 425–430 (1995).
